# RPS15 interacted with IGF2BP1 to promote esophageal squamous cell carcinoma development via recognizing m^6^A modification

**DOI:** 10.1038/s41392-023-01428-1

**Published:** 2023-06-02

**Authors:** Yahui Zhao, Yang Li, Rui Zhu, Riyue Feng, Heyang Cui, Xiao Yu, Furong Huang, Ruixiang Zhang, Xiankai Chen, Lei Li, Yinghui Chen, Yuhao Liu, Jinhua Wang, Guanhua Du, Zhihua Liu

**Affiliations:** 1 State Key Laboratory of Molecular Oncology, National Cancer Center, National Clinical Research Center for Cancer, Cancer Hospital, Chinese Academy of Medical Sciences and Peking Union Medical College, Beijing, 100021 China; 2 Department of Oncology, Cancer Institute, Peking University Shenzhen Hospital, Shenzhen Peking University-Hong Kong University of Science and Technology (PKU-HKUST) Medical Center, Shenzhen, 518035 China; 3 Department of Thoracic Surgery, National Cancer Center/National Clinical Research Center for Cancer/Cancer Hospital, Chinese Academy of Medical Sciences & Peking Union Medical College, Beijing, 100021 China; 4 Department of Radiation Oncology, National Cancer Center/National Clinical Research Center for Cancer/Cancer Hospital & Shenzhen Hospital, Chinese Academy of Medical Sciences and Peking Union Medical College, Shenzhen, 518116 China; 5 School of Life Sciences, Tsinghua University, Beijing, 100084 China; 6 Key Laboratory of Drug Target Research and Drug Screen, Institute of Materia Medica, Chinese Academy of Medical Science and Peking Union Medical College, Beijing, 100050 China

**Keywords:** Prognostic markers, Drug development

## Abstract

Increased rates of ribosome biogenesis have been recognized as hallmarks of many cancers and are associated with poor prognosis. Using a CRISPR synergistic activation mediator (SAM) system library targeting 89 ribosomal proteins (RPs) to screen for the most oncogenic functional RPs in human esophageal squamous cell carcinoma (ESCC), we found that high expression of RPS15 correlates with malignant phenotype and poor prognosis of ESCC. Gain and loss of function models revealed that RPS15 promotes ESCC cell metastasis and proliferation, both in vitro and in vivo. Mechanistic investigations demonstrated that RPS15 interacts with the K homology domain of insulin-like growth factor 2 mRNA-binding protein 1 (IGF2BP1), which recognizes and directly binds the 3′-UTR of *MKK6* and *MAPK14* mRNA in an m^6^A-dependent manner, and promotes translation of core p38 MAPK pathway proteins. By combining targeted drug virtual screening and functional assays, we found that folic acid showed a therapeutic effect on ESCC by targeting RPS15, which was augmented by the combination with cisplatin. Inhibition of RPS15 by folic acid, IGF2BP1 ablation, or SB203580 treatment were able to suppress ESCC metastasis and proliferation *via* the p38 MAPK signaling pathway. Thus, RPS15 promotes ESCC progression *via* the p38 MAPK pathway and RPS15 inhibitors may serve as potential anti-ESCC drugs.

## Introduction

Esophageal cancer ranks seventh in incidence and sixth in total mortality worldwide with dismal clinical outcomes, being responsible for over 500,000 deaths in 2018.^1^ Esophageal squamous cell carcinoma (ESCC) is the predominant histological subtype of esophageal cancer, and has an aggressive clinical course and poor prognosis.^2^ ESCC is a common aggressive malignancy with unclear molecular and prognostic biomarkers. The rapid proliferation and metastasis of tumor cells are the main characteristics of this disease. The limited availability of targeted therapies and insufficient clinical management demonstrate the need for further investigations regarding the molecular mechanisms of ESCC pathogenesis in order to ultimately improve treatment outcomes.

Ribosomes are conserved molecular machines that mediate translation, with the levels of ribosome proteins being tightly linked to cellular growth rates. Increased rates of ribosome biogenesis result in aberrant increases in nucleolar size and number, which have been recognized as hallmarks of many cancers and are associated with poor prognosis.^3^ The ribosome is composed of numerous ribosomal proteins (RPs) and nucleic acids, and translation is a complex cellular process. Eukaryotes have 80S ribosomes consisting of 40S subunits with 18S rRNA and ~33 small subunit ribosomal proteins (RPSs) and 60S subunits with 5S rRNA, 28S rRNA, a 5.8S subunit, and ~46 large subunit ribosomal proteins (RPLs).^4,5^ The increased number of ribosomes is not only necessary, but also sufficient to drive the transformation of malignant tumors. For instance, RPL5, RPS3, RPS6, RPS8, and RPS12 are highly expressed in colorectal cancer (CRC) tissues compared to that of normal tissues. Furthermore, elevated levels of RPS8, RPL12, RPL23a, RPL27, and RPL30 mRNAs have been detected in human liver cancer tissues.^6^ Recent discoveries of RPL15 overexpression in circulating breast cancer tumor cells,^7^ and mutations of RPL5,^8^ RPL10,^9^ and RPS15^10,11^ in leukemia have inspired interest regarding how these RPs are associated with oncogenesis and disease progression. Deregulation of RPL15 expression enhances the translation of RPs and E2F pathway proteins to promote breast cancer metastasis, while mutations in RPs, including RPL10 and RPS15, also provide a potential oncogenic mechanism. Since the lack of functional study of RPs in ESCC, we constructed a CRISPR synergistic activation mediator (SAM) system library targeting 89 RPs, including 54 RPLs and 35 RPSs, in two ESCC-derived cell lines to screen for the most oncogenic functional ribosomal proteins. RPS15 is a vital ribosomal protein of the 40 s small subunit,^12^ mediating interactions between the 40S and 60S ribosomal subunits and interacting with mRNA and tRNA.^13^ Therefore, RPS15 is an essential component of mRNA translation and ribosomal biogenesis. However, despite this insight into the role of RPS15 in protein translation and tumor progression, the mechanisms of translational alteration and downstream regulation remain undefined.

N^6^-methyladenosine (m6A) is the most common, abundant, and conserved transcriptional modification, which mainly occurs in the messenger RNA (mRNA) of eukaryotes.^14^ The m^6^A modification is installed by m^6^A methyltransferases (Writer), recognized by m^6^A binding proteins (Reader), and removed by demethylases (eraser). There is increasing evidence that m^6^A RNA methylation greatly affects RNA metabolism and participates in the pathogenesis of many diseases, including cancer progression.^15,16^ The function of m^6^A-modified RNA are mediated mostly through readers, which mainly include the YTH domain family proteins (YTHDF1, YTHDF2, YTHDF3, and YTHDC1),^17,18^ and insulin-like growth factor 2 mRNA-binding proteins (IGF2BPs) family (IGF2BP1, IGF2BP2, and IGF2BP3).^19^ However, different readers have a different biological effect on downstream. In the YTH domain family, YTHDF1 enhances mRNA translation and protein synthesis by binding to initiation factors.^20^ YTHDF2 induces the degradation of transcripts by selectively binding to m^6^A-modified mRNA and recruiting it to mRNA decay sites.^21^ YTHDF3 enhances RNA translation by interacting with YTHDF1 and promotes RNA degradation by binding with YTHDF2.^18,22^ In addition, IGF2BPs promote RNA expression by enhancing RNA stability.^23–25^

Functional and mechanistic studies of RPS15 will not only provide insight into the pathogenesis of ESCC, but also provide an opportunity to better understand how protein translation may play a key role in cancer cell metastasis. Therefore, we used ribosome profiling and deep transcriptome analysis of aberrantly expressed RPS15 to define the role of ribosomal changes. Importantly, we demonstrated that RPS15 promoted downstream translation through binding with IGF2BP1, an m^6^A methylation reader. Our work revealed how deregulated RP expression could combine with epigenetic regulation to modulate tumor metastasis. This will help identify candidate targets of RPS15 that are involved in ESCC progression.

## Results

### High RPS15 expression correlated with ESCC metastasis and poor prognosis

**Fig. 1:**
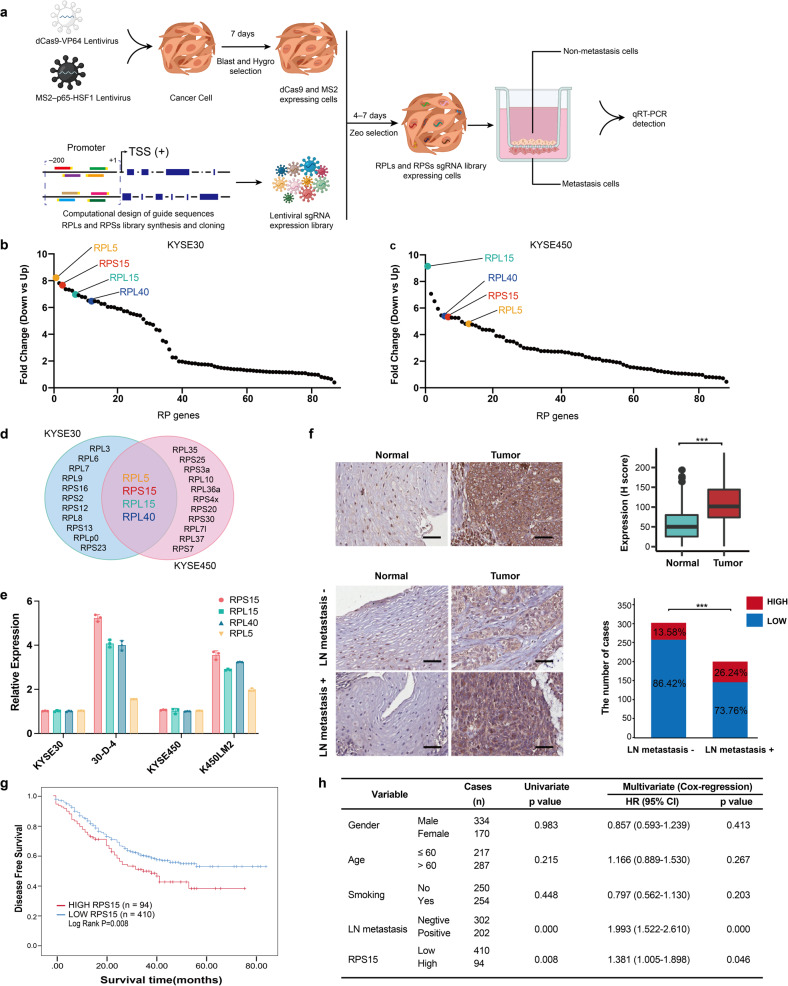
High RPS15 expression correlates with malignant phenotype and poor prognosis in ESCC. **a** Schematic created with BioRender.com demonstrating the screening strategy for pro-metastatic ribosome proteins (RPs). **b**, **c** qRT-PCR analysis of fold changes in gene expression of 89 RPs between metastatic and non-metastatic of KYSE30 cells (**b**) and KYSE450 cells (**c**). Data were presented as mean ± SD; *n* = 3. Two-tailed *t*-tests. **d** Venn diagram showing the RPs upregulated in metastasis of KYSE30 and KYSE450 cells. **e** qRT-PCR analysis of fold changes of four RPs expression in highly metastatic ESCC cell lines 30-D-4 and K450LM2. Data were presented as mean ± SD; *n* = 3. Two-tailed *t*-tests. **f** Representative images (left) and the statistical analysis (right) of TMA-based IHC showing higher RPS15 expression in tumor tissue (upper panel). The expression of RPS15 is correlated with lymph node metastasis (lower panel). Scale bars, 50 μm. The data were analyzed using paired two-tailed *t*-test and Fisher exact test and are presented as mean ± SD. *P < 0.05 and ***P < 0.001. **g** Kaplan–Meier survival curve showing that high expression of RPS15 correlated with lower disease-free survival in patients with ESCC (*P* < 0.05). **h** Multivariate Cox regression analysis of factors associated with survival of patients with ESCC

To identify novel ribosome proteins in promoting ESCC metastasis, we designed a CRISPR SAM system library targeting 89 RPs, including members of both the RPL and RPS families. ESCC cell lines KYSE30 and KYSE450 expressing nuclease-dead Cas9 (dCas9) and male sterile 2 (MS2) enzymes were infected with lentiviruses harboring the single guide RNA (sgRNA) library (Supplementary Table S1) and then selected using puromycin for 10 days. The KYSE30 and KYSE450 cells were then resuspended in serum-free medium and seeded into the upper Boyden Chamber precoated with Matrigel (Fig. 1a). After 48 h of incubation, the cells were collected from the upper (non-metastatic cells) and lower (metastasis cells) chambers, RNA was extracted from both groups of cells, and mRNA expression of the 89 RPs was detected (Fig. 1b, c). We subsequently focused on the four significantly upregulated RPs that overlapped in the metastatic groups of the two ESCC cell lines and were enriched in the metastatic cells recovered from the lower chamber (Fig. 1d). To further validate the potential role of these four RPs in ESCC metastasis, we measured their expression levels in two highly metastatic esophageal cancer cell lines, 30-D-4 and K450LM2 cells.^26–28^ Real-time quantitative reverse transcription polymerase chain reaction (qRT-PCR) results showed that RPS15 significantly upregulated in metastatic cells compared with that in the parental cells (Fig. 1e). Next, we performed immunohistochemical (IHC) staining of RPS15 in 504 ESCC specimens. IHC analysis of tissue microarrays (TMAs) revealed higher expression of RPS15 in tumors compared with that in normal tissue (*t*-test, *P* = 6.00E − 45; Fig. 1f upper panel). Moreover, the increased RPS15 expression was significantly correlated with lymph node metastasis (Fisher exact test, *P* = 0.018; Fig. 1f lower panel). Finally, we analyzed the impact of RPS15 expression on the survival time of patients with ESCC in our sample set. The Kaplan–Meier curves showed that high RPS15 expression in patients with ESCC significantly correlated with poor prognosis (*P* = 0.008; Fig. 1g). Furthermore, multivariate Cox regression analysis showed that high RPS15 expression was an independent risk factor for metastasis and poor prognosis for patients with ESCC (Fig. 1h).

### RPS15 promoted ESCC metastasis and proliferation in vitro

**Fig. 2:**
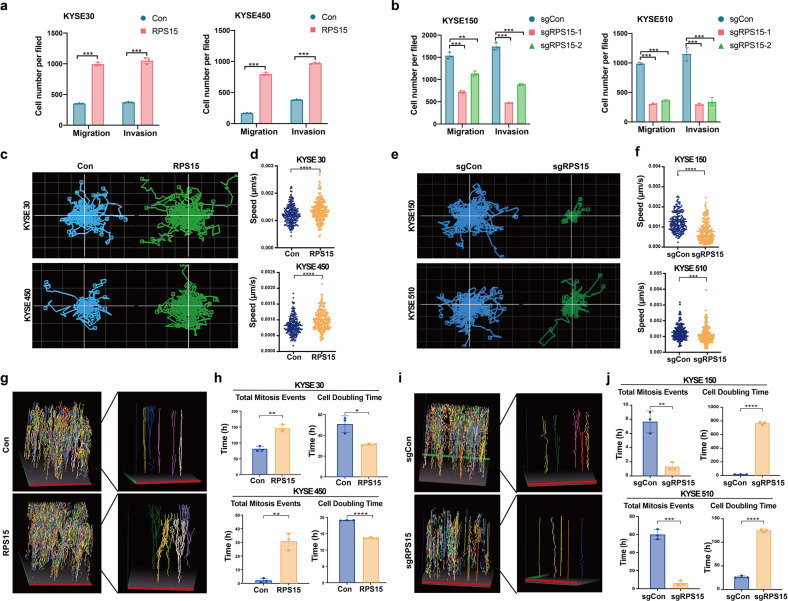
RPS15 promoted ESCC metastasis and proliferation in vitro. **a** Statistical analyses of Boyden Chamber migration and invasion assays for KYSE30 cells (left) and KYSE450 cells (right) stably transfected with control vector or RPS15-overexpression vector. **b** Statistical analyses of Boyden Chamber migration and invasion assays for KYSE150 cells (left) and KYSE510 cells (right) with or without RPS15 knockout. **c**, **d** Random motility displacement (**c**) and track speed (**d**) of each cell type in the RPS15 overexpression and control group in KYSE30 (upper) and KYSE450 (lower) cells. **e**, **f** Random motility displacement (**e**) and track speed (**f**) of each cell type in the sgRPS15 and sgCon groups in KYSE150 (upper) and KYSE510 (lower) cells. The experiments were performed by using Livecyte (Phasefocus, UK) and data were analyzed using two-tailed *t*-tests. **g** The three-dimensional (3D) representation and close-up view of the tracking of each cell type in RPS15-overexpressing and control groups of KYSE30 cells. **h** Total mitosis events and cell-doubling time of each cell type in RPS15-overexpressing and control groups in KYSE30 cells (upper) and KYSE450 cells (lower). **i** The 3D representation and close-up view of the tracking of each cell type in the sgRPS15 and sgCon groups of KYSE150 cells. **j** Total mitosis events and cell-doubling time of each cell type in the sgRPS15 and sgCon groups in KYSE150 cells (upper) and KYSE510 cells (lower). Data were analyzed using unpaired *t*-tests. *P < 0.05, **P < 0.01, and ***P < 0.001

To verify the role of RPS15 in ESCC, we measured RPS15 expression levels in nine ESCC cell lines and the human esophageal epithelial cell line Het1A using Western blot. The results showed that *RPS15* expression was relatively higher in KYSE150 and KYSE510 cells, but lower in KYSE30 and KYSE450 cells (Supplementary Fig. S1a). Thus, we established the stable overexpression of RPS15 in KYSE30 and KYSE450 cells and knockout of RPS15 (sgRPS15) in KYSE150 and KYSE510 cells to determine the role of RPS15 on cancer cell metastasis and growth. The Boyden Chamber migration and invasion assays were used to investigate the role of RPS15 in ESCC metastasis in vitro. As shown in Fig. 2a, b and Supplementary Fig. S1b, c, overexpression of RPS15 substantially increased the migration and invasion abilities of KYSE30 and KYSE450 cells, whereas knockout of RPS15 in KYSE150 and KYSE510 cells resulted in a significant decrease in these abilities. We also used a ptychographic QPI label-free imaging technique to obtain the visualization results, which were used to measure cell movement. We found that overexpression of RPS15 increased the track speed and movement ability of ESCC cells (Fig. 2c, d), while RPS15 depletion resulted in a loss of these abilities (Fig. 2e, f). As for cell proliferation, the morphological measurements, including cell-doubling time and total mitosis events, were used to compare differences between the RPS15 overexpression group and the RPS15 knockout group of ESCC cells. The results showed that RPS15 overexpression promoted cell mitosis events and reduced cell-doubling time (Fig. 2g, h), while RPS15 knockout had the opposite effect (Fig. 2i, j). Consistent with these results, we also found that overexpression of RPS15 significantly increased the proliferation abilities of KYSE450 cells (Supplementary Fig. S2a, b). Furthermore, we also inhibited the expression of RPS15 in KYSE150 and KYSE510 cells using small interfering RNA (siRNA). The siRNA results demonstrated that cell proliferation abilities were significantly decreased when *RPS15* was downregulated in KYSE150 and KYSE510 cells (Supplementary Fig. S2c–f).

### The effects of RPS15 on ESCC development in vivo

**Fig. 3:**
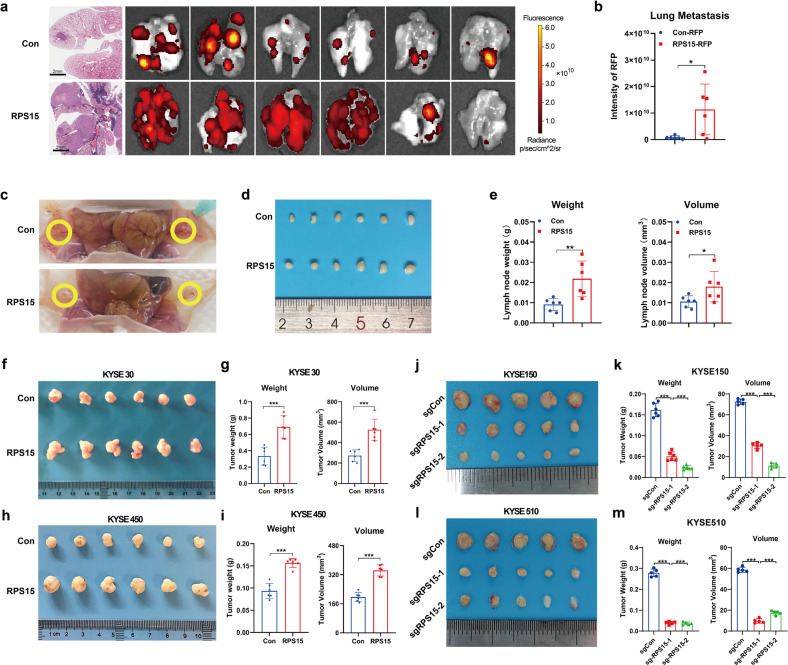
The effects of RPS15 in ESCC development in vivo. **a**, **b** A lung metastatic mouse model was established using RPS15-RFP overexpressed KYSE30 cells, which were injected intravenously into 5-week-old male SCID beige mice *via* the tail vein (*n* = 6 per group). Twelve weeks later, RFP imaging (**a**) and quantification of RFP intensity of lung metastases (**b**) were recorded. Data were analyzed using unpaired t-tests. *P < 0.05, **P < 0.01, and ***P < 0.001. **c–e** A lymphatic metastasis mouse model of ESCC was established by injecting RPS15-overexpressing KYSE30 cells into the left footpad of mice (*n* = 6 per group). The yellow circle indicates the metastatic popliteal lymph node (**c**), macroscopic appearance (**d**), and quantification of the weight and volume (**e**) of metastatic popliteal lymph nodes. **f**–**i** Macroscopic appearance (**f**), weights, and volume (**g**) of xenografts of RPS15-overexpressing KYSE30 cells (*n* = 6 per group, upper). Macroscopic appearance (**h**), weights, and volume (**i**) of xenografts of RPS15-overexpressing KYSE450 cells (*n* = 6 per group, upper). **j**–**m** Macroscopic appearance (**j**, **l**), weights, and volume (**k**, **m**) of KYSE150 cell xenografts with or without RPS15-knockout (w/o RPS15 knockout, *n* = 5 per group) and KYSE510 cell xenografts (w/o RPS15 knockout, *n* = 5 per group), respectively. Data were analyzed using unpaired t-tests. *P < 0.05, **P < 0.01, and ***P < 0.001

To investigate whether RPS15 had an impact on tumor metastasis in vivo, we used a Red fluorescent protein (RFP)-based lung metastatic mouse model. RFP imaging and quantification of the RFP intensity of lung metastases in each animal showed that stable overexpression of RPS15 in KYSE30 cells resulted in substantially increased lung metastasis (*t*-test, *P* < 0.05; Fig. 3a, b). In the popliteal lymph node metastasis model, we found that overexpression of RPS15 in ESCC cells significantly increased lymphatic metastasis, as indicated by lymph node weights and volumes (*t*-test, *P* < 0.05; Fig. 3c–e). To verify the oncogenic role of RPS15 in vivo, we established a subcutaneous transplantation tumor model in BALB/c-nude mice using RPS15 stable overexpression (RPS15) and RPS15 knockout (sgRPS15) ESCC cell lines. At 4 weeks post-transplantation, the mice were sacrificed, and the tumors were excised and analyzed. The results showed that the tumor growth rate (tumor weight and tumor volume) of the RPS15 group was significantly higher compared with that of the control group (*t*-test, *P* < 0.001; Fig. 3f–i), while the growth rate of the sgRPS15 group was slower compared with that of the sgCon group (*t*-test, *P* < 0.001; Fig. 3j–m). Taken together, these results indicate that high expression of RPS15 may be a special contributing event in the development of ESCC in vivo.

### RPS15 activated the p38 mitogen-activated protein kinase (MAPK) and E2F pathways

**Fig. 4:**
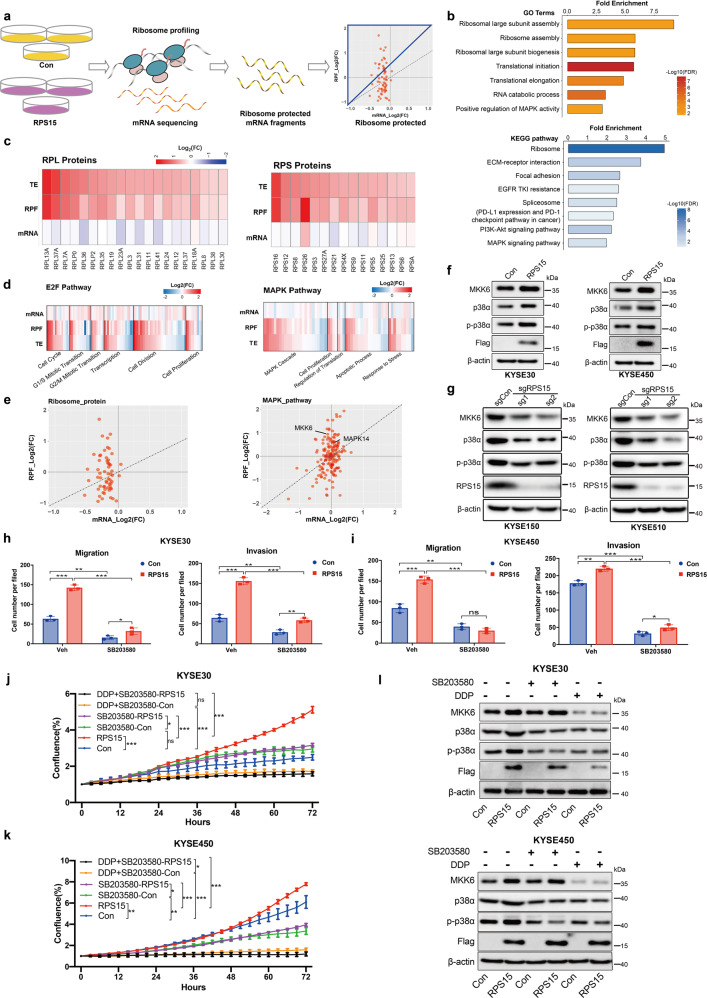
RPS15 overexpression promotes the translation of core E2F and p38 MAPK pathway proteins. **a** Schematic illustrating the ribosome profiling of KYSE30 cells (Con) and RPS15-overexpressing KYSE30 cells (RPS15). **b** GO analysis (upper) and KEGG-pathway analysis (lower) of transcripts preferentially bound by ribosomes in the RPS15 group. The most enriched ribosomal/translational GO gene sets and associated FDR values are shown, which were mainly enriched in translation and MAPK signaling pathways. **c** Heat map of the log2 fold-change of the RPS15 group relative to that of the control group for ribosome protein genes for mRNA expression, ribosome-protected fragments (RPF), and translation efficiency (TE). **d** Heat map of the log2 fold-change of the RPS15 group relative to that of the control for each gene of the E2F pathway and p38 MAPK pathway target-gene set for mRNA expression, RPF, and TE. Genes were categorized according to their GO biological process functions in the relative order of the TE fold-change. **e** Scatter plot of the translational efficiency of individual RP genes (right panel) and the hallmark p38 MAPK-target gene set transcripts (left panel). The x-axis represents the log2 fold-change in RNA-seq and the y-axis represents the log2 fold-change in ribosome profiling. **f** Expression of the hallmark p38 MAPK-target gene set in RPS15-overexpressing KYSE30 cells (left) and KYSE450 cells (right) determined by Western blot. **g** Western blot analysis results of expression of the hallmark p38 MAPK-target gene set in KYSE150 cells (left) and KYSE510 cells (right) with or without RPS15 knockout. **h**, **i** Statistical analyses of Boyden Chamber migration and invasion assays for KYSE30 cells (**h**) and KYSE450 cells (**i**) with stably transfected control vector or RPS15-overexpressing vector with or without SB203580 treated for 24 h. The statistical analysis results are shown. **j** Growth curves measured using Incucyte live-cell analyses of KYSE30 cells stably transfected with control vector (blue) or RPS15-overexpressing vector (red) and treated with SB203580 or SB203580 combined DDP for 72 h. **k** Growth curves measured using Incucyte live-cell analyses of KYSE450 cells stably transfected with control vector (blue) or RPS15-overexpressing vector (red) and treated with SB203580 or SB203580 combined DDP for 72 h. Data were analyzed using unpaired *t*-tests. *P < 0.05, **P < 0.01, and ***P < 0.001. **l** Western blot analysis to detect expression of the hallmark MAPK-target gene set in KYSE30 cells (upper) and KYSE450 cells (lower) stably transfected with control vector or RPS15-overexpressing vector and treated with SB203580 or DDP for 2 days. Con control, Veh vehicle

As components of the ribosome, RPs are reported to have a highly coordinated expression to ensure the fidelity of ribosome subunit biogenesis and assembly.^3^ Thus, the altered expression of RPS15 may alter ribosome translation efficiency, either globally or within specific subsets of mRNAs.^29^ Accordingly, we performed ribosomal profiling of KYSE30 cells with stable overexpression of RPS15 (RPS15) and the corresponding negative control (Con) cells to determine the global ribosome occupancy of mRNA transcripts, to identify changes in translational preference, and to calculate the translational efficiency for any given transcript (ratio of ribosome-bound mRNA to total mRNA)^30^ (Fig. 4a). Gene ontology (GO) enrichment analysis was performed for genes whose transcripts were preferentially bound by ribosomes in the RPS15 samples. Genes within GO terms for translation and positive MAPK regulation were significantly enriched in RPS15 overexpression cells as indicated by a false discovery rate (FDR) <0.01 (Fig. 4b). Moreover, Kyoto Encyclopedia of Genes and Genomes (KEGG) pathway enrichment analysis showed that ribosomal process and the p38 MAPK signaling pathway (fold enrichment = 2.18, *P* < 0.01), especially MKK6 and MAPK14, exhibited significant enrichment of transcripts preferentially bound to the ribosome in RPS15 overexpression cells (Fig. 4b). Ribosome-protected transcripts of RPs were markedly increased in RPS15 overexpression cells, including both large subunits (mean 1.75-fold in the top 20) and small subunits (mean 1.70-fold in the top 15) (Fig. 4c). These results indicate that overexpression of RPS15 could give rise to coordinated upregulation of translation efficiency for core RPs. Inspired by the increased proliferation rate phenotype of RPS15 overexpression, we evaluated the changes in ribosome-protected fragments (RPF) and translation efficiency (TE) of genes in the E2F pathway. The results demonstrated that the bulk of genes in the E2F pathway, especially those involved in cell cycle and cell division, exhibited higher TE in RPS15 overexpression cells compared with that in the control cells (Fig. 4d, e). Notably, the efficiency of genes belonging to the p38 MAPK pathway, which mainly affects the MAPK cascade and cell proliferation, was significantly increased in RPS15 overexpression cells compared with that in the control cells, especially MKK6 and p38. Furthermore, we performed a Western blot to investigate the molecular mechanisms underlying the effects of RPS15 on the MAPK pathway. As shown in Fig. 4f, g, the hallmark p38 MAPK pathway genes MKK6, p38, and p-p38 were simultaneously upregulated upon RPS15 overexpression in KYSE30 and KYSE450 cells and suppressed in RPS15-depleted ESCC cells. Taken together, these findings demonstrated that overexpression of RPS15 augmented global protein translation, with a selectively enhanced impact on the translation of transcripts encoding members of the E2F and MAPK pathways.

### The p38 MAPK inhibitor SB203580 mimicked the effects of RPS15 ablation

To test the role of p38 MAPKs in the ESCC malignant phenotype regulated by RPS15, we selected SB203580, a small-molecule inhibitor of p38 MAPK, to inhibit the activation of the p38 MAPK pathway. Boyden Chamber migration and invasion assays were performed to investigate whether the p38 MAPK pathway affected migration and invasion in RPS15-overexpressing ESCC cells. The Boyden Chamber results indicated that overexpression of RPS15 substantially increased the migration and invasion abilities of ESCC cells (Fig. 4h, i and Supplementary Fig. S3a, b), whereas inhibition of the p38 MAPK pathway resulted in a significant decrease in these abilities, especially in the RPS15 overexpression group. In parallel, we found, based on growth curves, that SB203580 suppressed the growth-promoting effect of RPS15 overexpression in KYSE30 and KYSE450 cells. As SB203580 treatment alone inhibited ESCC cell proliferation to a moderate degree, we examined the therapeutic efficacy of the combination of SB203580 and DDP. The combination of 8 μM SB203580 and 0.5 μg/ml DDP resulted in inhibition of the proliferation of RPS15-overexpressing ESCC cells by as much as 69% for KYSE30 cells and 84.6% for KYSE450 cells compared with that of the controls, which represented a further inhibition of 42% for KYSE30 cells and 35.33% for KYSE450 cells compared with that of SB203580 alone (Fig. 4j, k). Western blot analysis indicated that SB203580 and DDP significantly reduced the expression of the p38 MAPK pathway hallmark genes MKK6, p38, and p-p38 in ESCC cells (Fig. 4l). These data demonstrate that SB203580 exhibited a therapeutic effect on ESCC by blocking RPS15 *via* the p38 MAPK pathway with the effect being augmented by the combination treatment with DDP.

### RPS15 interacted with the K homology (KH) domain of IGF2BP1

**Fig. 5:**
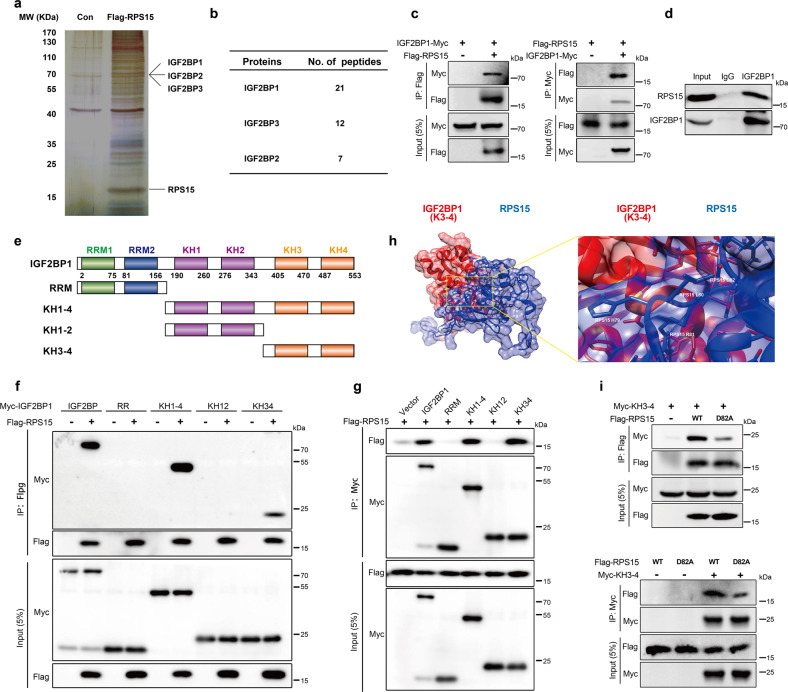
RPS15 interacted with the K homology (KH) domain of IGF2BP1. **a** Silver staining and MS analysis of FLAG-RPS15 IP. The most significant genes are shown. **b** Number of unique peptide hits for RPS15, IGF2BP1, IGF2BP2, and IGF2BP3. **c** Co-IP assay of RPS15 and IGF2BP1 in vitro. Flag-RPS15 and Myc-IGF2BP1 plasmids were co-transfected into HEK 293 T cells. The immunoprecipitates were analyzed by Western blot using anti-FLAG and anti-Myc antibodies. **d** Co-IP assay of RPS15 and IGF2BP1 in vivo. The immunoprecipitates were analyzed by Western blot using anti-IGF2BP1 antibodies. **e** Diagram of the different domains of the IGF2BP1 constructs. **f**, **g** Co-IP assay to assess the interaction between RPS15 and different domains of IGF2BP1 in vitro. Flag-RPS15 and Myc-RRM, Myc-KH1-4, Myc-KH1-2, and Myc-KH3-4 plasmids were transfected into HEK 293 T cells. The immunoprecipitates were analyzed by Western blot using anti-Flag (**f**) and anti-Myc antibodies (**g**). **h** Molecular docking model of RPS15 interacting with the KH3-4 domain of IGF2BP1 (https://cluspro.bu.edu/). **i** Co-IP assay to identify the amino acid sites of RPS15 binding to IGF2BP1. The immunoprecipitates were analyzed by Western blot using an anti-Flag antibody (upper) and anti-Myc antibody (lower)

To investigate the underlying mechanism and the cofactors of RPS15 that activated the p38 MAPK pathway, we performed an immunoprecipitation assay using anti-flag magnetic beads and ESCC cells stably overexpressing Flag-tagged RPS15 or Flag-tagged control cells. Mass spectrometry (MS) analysis was used to identify the proteins that potentially interacted with RPS15 (Fig. 5a). Among the proteins identified by MS, we focused on those of the IGF2BP family (IGF2BP1/2/3) (Fig. 5b), which is a new family of m^6^A readers that protect m^6^A-modified mRNAs from decay.^19^ To validate the interaction between RPS15 and IGF2BPs, we first co-transfected Flag-RPS15 and Myc-IGF2BPs into HEK293T cells and then performed co-immunoprecipitation (co-IP) using anti-flag magnetic beads and anti-myc agarose beads, respectively. The co-IP analysis confirmed that RPS15 could specifically and directly bind to IGF2BP1, but not to IGF2BP2 or IGF2BP3 (Fig. 5c and Supplementary Fig. S4a, b). The association between endogenous RPS15 and IGF2BP1 was validated with co-IP assays using KYSE450 cells and an anti-IGF2BP1 antibody (Fig. 5d). IGF2BP1 consists of two N-terminal RNA recognition domains (RRM1 and RRM2) and four C-terminal heterogeneous ribonucleoprotein (hnRNP) KH domains (KH1–KH4).^31^ To further map the association between RPS15 and IGF2BP1, several plasmids expressing truncated IGF2BP1 proteins were constructed to investigate which domain is bound to RPS15. We truncated IGF2BP1 protein into four components, which are the RRM domain, KH1-4 domain, KH1-2 domain, and KH3-4 domain (Fig. 5e). Co-IP assays showed that RPS15 selectively bound to the KH3-4 domain of IGF2BP1, not RRM and KH1-2 (Fig. 5f, g). Additionally, a molecular docking model (ClusPro) was used to predict the binding of RPS15 residues to IGF2BP1 (Fig. 5h).^32^ Combined with the results from ClusPro and the hydrophobic score of amino acids from the ProtScale database, we constructed five RPS15 mutants to perform the co-IP assay, which includes Q24A, D82A, P87A, M93A, and P109A. Co-IP assays showed that mutating the D82 residue to A82 destroyed the binding of RPS15 to IGF2BP1 (Fig. 5i and Supplementary Fig. S4c, d). The co-IP analysis confirmed that RPS15 could directly bind to IGF2BP1, RPS6, RPL11, and RPL23A, which further demonstrated that RPS15, IGF2BP1, and the MKK6/MAPK14 mRNA exist in the properly assembled ribosome (Supplementary Fig. S4e). In summary, our results indicated that RPS15 interacted with the KH3-4 domain of IGF2BP1 directly, which augmented global protein translation, with a selectively enhanced impact on the translation of transcripts of MAPK pathway genes.

### RPS15 regulated the p38 MAPK pathway through the direct binding of IGF2BP1 to *MAPK14* and *MKK6* mRNA transcripts

**Fig. 6:**
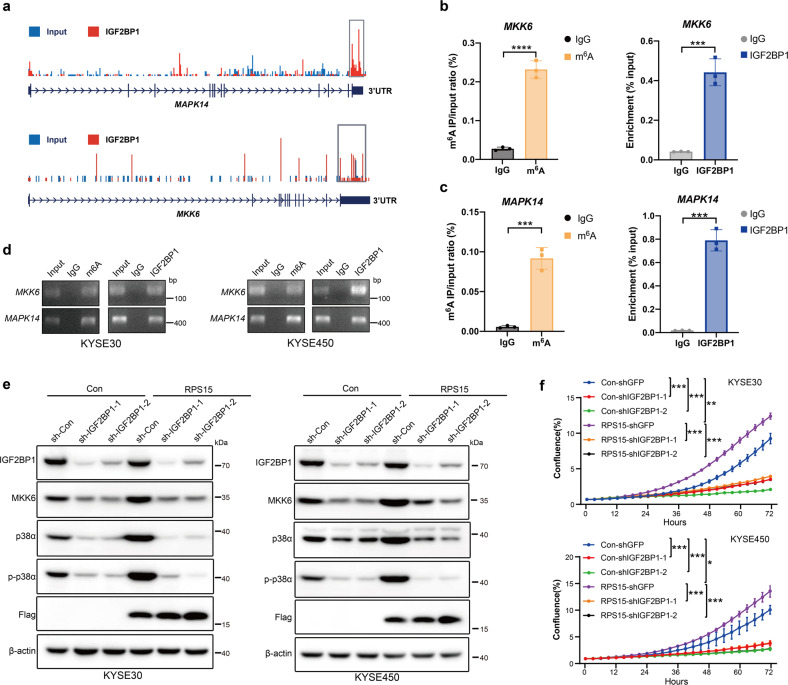
IGF2BP1 binds the 3′-UTR of MKK6 and MAPK14 in an m6A-dependent manner. **a** Distribution of m^6^A peaks of IGF2BP1 RIP-seq data across *MAPK14* and *MKK6* mRNA transcripts. **b** Enrichment of m6A modification in 3′-UTR region of *MKK6* with Flag-tagged IGF2BP1 in KYSE30 cells (left); RIP-qPCR showing the binding of IGF2BP1 to the 3′-UTR region of *MKK6* (right). **c** Enrichment of m6A modification in 3′-UTR region of *MAPK14* with Flag-tagged IGF2BP1 in KYSE30 cells (left); RIP-qPCR showing the binding of IGF2BP1 to the 3′-UTR region of *MAPK14* (right). **d** Agarose gel electrophoresis showing the binding of IGF2BP1 to the 3′-UTR region of *MKK6* and *MAPK14* in KYSE30 cells (left) and KYSE450 cells (right). **e** Western blot detected protein expression of the hallmark p38 MAPK-target gene set in RPS15-overexpressing KYSE30 cells (left) and KYSE450 cells (right) with or without IGF2BP1 knockdown. **f** Growth curves measured using Incucyte live-cell analyses of KYSE30 cells (upper) and KYSE450 cells (lower) stably transfected with control vector (blue) or RPS15-overexpressing vector (purple) and treated with IGF2BP1 knockdown or without IGF2BP1 knockdown for 72 h. Data were analyzed using unpaired t-tests. *P < 0.05, **P < 0.01, and ***P < 0.001

A previous study reported that the KH3-4 di-domain is indispensable for m^6^A recognition and binding.^19^ To determine whether IGF2BP1 regulates the p38 MAPK pathway in the form of m^6^A modification recognition, IGF2BP1 RNA immunoprecipitation sequencing (RIP-seq) data from previous reports were re-analyzed to identify m^6^A peaks across *MAPK14* and *MKK6* mRNA transcripts.^19^ As shown in Fig. 6a, IGF2BP1 binding peaks accumulated across *MAPK14* (encoded p38 protein) and *MKK6* transcripts, especially in the 3′-UTR, indicating that IGF2BP1 bound directly to *MAPK14* and *MKK6* mRNA transcripts. By conducting RIP and gene-specific m^6^A assays (Fig. 6b–d and Supplementary Fig. S5a, b), we confirmed the m^6^A modification and IGF2BP1 binding in the same region of *MKK6* and *MAPK14*. Furthermore, we detected the expression of MKK6 and p38 in IGF2BP1-ablated ESCC cells and Western blot analysis showed that the knockdown of IGF2BP1 in RPS15-overexpressing ESCC cells diminished the upregulating effect of RPS15 on MKK6 and p38 expression (Fig. 6e). To further investigate the function of IGF2BP1, we inhibited its expression in RPS15-overexpressing ESCC cells using its short hairpin RNA (shRNA) expressed by a lentivirus vector. Growth curves revealed that the overexpression of RPS15 promoted the proliferation of the ESCC cells, while the knockdown of IGF2BP1 inhibited this effect on ESCC growth (Fig. 6f). Taken together, our data demonstrate that RPS15 could promote the translation of *MAPK14* and *MKK6* mRNA, which were directly bound and recognized in an m^6^A dependent manner by the KH3-4 domain of IGF2BP1, which then enhanced p38 and MKK6 TE and promoted the proliferation and metastasis of ESCC cells.

### Folic acid suppressed ESCC development by targeting RPS15

**Fig. 7:**
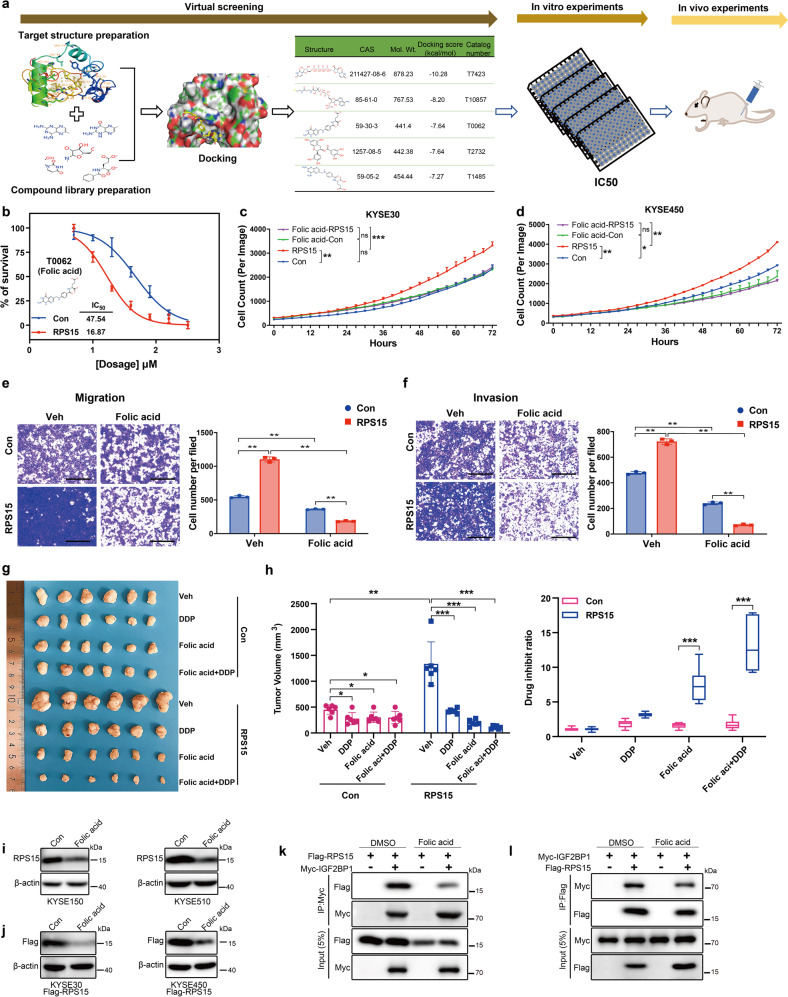
Folic acid suppresses ESCC metastasis and proliferation by targeting RPS15. **a** Typical workflow of docking-based virtual screening of 3218 FDA-approved drugs. **b** Inhibition curves (measured using CCK8 assays) of KYSE30 cells stably transfected with control vector (blue) or RPS15-overexpressing vector (red) treated with folic acid (T0062) for 24 h. **c**, **d** Growth curves measured by Incucyte live-cell analyses of KYSE30 cells (**c**) and KYSE450 cells (**d**) stably transfected with control vector (blue) or RPS15-overexpressing vector (red) treated with folic acid (pink) or without folic acid (green) for ~72 h. **e**, **f** Representative image (left panel) and statistical analyses (right panel) of Boyden Chamber migration (**e**) and invasion (**f**) assays of KYSE30 cells stably transfected with control vector or RPS15-overexpressing vector treated with or without folic acid for 24 h. Scale bar: 500 μm. **g**, **h** Macroscopic appearance (**g**) and tumor volume and drug inhibition ratio (**h**) of xenografts of RPS15-overexpressing KYSE30 cells (*n* = 6 per group). Data were analyzed using unpaired *t*-tests. *P < 0.05, **P < 0.01, and ***P < 0.001. Con control, Veh vehicle. **i**, **j** Western blot detected protein expression of RPS15 in KYSE150 and KYSE510 (**i**) (high expression of RPS15), and KYSE30 and KYSE450 cells stably transfected with Flag-RPS15-overexpressing vector (**j**), with or without folic acid (T0062, 10 μM) treatment for 24 h. **k**, **l** Co-IP assay of RPS15 and IGF2BP1 in vitro. Flag-RPS15 and Myc-IGF2BP1 plasmids were co-transfected, and then treated with or without folic acid (T0062, 10 μM). The immunoprecipitates were analyzed by co-IP assay using anti-Myc (**k**) and anti-Flag (**l**) antibodies

To identify a small molecular inhibitor that could specifically target RPS15, we used the nuclear magnetic resonance (NMR)-resolved crystal structure of RPS15 from the human 40S subunit [Protein Data Base (PDB) ID: 6G4W] as the starting structure for our models. Protein Preparation Wizard was then used to optimize the protein structure. Schrodinger sitemap was used to identify three potential small-molecule binding pockets in the RPS15 protein structure, and the one with the highest grid-based docking program Glide score was defined as the binding pocket for Receptor Grid Generation. For the ligand library, we screened the marketed drug database, which originally contained 3218 compounds. Schrodinger-QikProp was used to predict the most extensive properties of drugs by calculating more than 20 physical descriptors. Finally, Schrodinger-LigPre was used to optimize all the small molecules. During the optimization process of the small molecules, Merck molecular force field (MMFF) was used; all the other parameters were default settings. All compounds during the entire virtual screening process were connected to the defined binding pocket in three steps. The first step was high flux screening (HTVS), in which 30% of the compounds were retained. The second step was standard precision (SP), which included 30% of the first step. The third step was extra precision (XP), in which 30% of the compounds in the second step were retained. The screening process of these drugs is shown in Fig. 7a. The first five screened drugs with high scores were selected (T7423, T10857, T0062, T2732, and T1485).

Combining the inhibition curves and growth curves, we found that folic acid (T0062) had a greater growth suppression effect on RPS15-overexpressing cells compared with that of other drugs (Fig. 7b–d and Supplementary Fig. S6a, b). To verify the inhibitory effect of folic acid on ESCC development, Boyden Chamber migration, and invasion assays were performed using KYSE30 cells stably transfected with RPS15-expressing vector or empty vector, then treated with or without folic acid. The Boyden Chamber assays showed that folic acid inhibited cell migration and invasion more efficiently in the RPS15 overexpression group compared with that of the empty-vector group (Fig. 7e, f). Furthermore, the results of an in vivo xenograft mouse model showed that folic acid significantly reduced tumor volume and inhibited ESCC growth, especially in the RPS15-overexpressing xenograft group. The inhibitory effect was stronger than that of cisplatin (DDP), which is the preferred drug for chemotherapy of esophageal cancer (Fig. 7g, h). Since folic acid treatment alone inhibited tumor growth at a moderate degree, we further examined the therapeutic efficacy of a combination of folic acid and DDP. Each mouse was intraperitoneally injected with 5 mg/kg folic acid and 5 mg/kg DDP. The growth of KYSE30 engraftments overexpressing RPS15 was inhibited by as much as 91.5% ± 0.025 compared with that of the controls, representing an additional 25.5% ± 0.066 inhibition compared with that of DDP alone (Fig. 7h). Besides, to verify the inhibitory effect of folic acid on RPS15, we detected the protein expression of RPS15 in ESCC cell lines treated with folic acid. Western blot analysis showed that folic acid could significantly decrease the protein expression level of RPS15 in KYSE150 and KYSE510 cells (high expression of RPS15) (Fig. 7i) or in the RPS15-overexpressing ESCC cells (Fig. 7j), but weak in KYSE30 and KYSE450 cells (low expression of RPS15) (Supplementary Fig. S6c, d). To further investigate the mechanism by which folic acid regulates RPS15 protein expression, we conducted a CHX pulse-chase assay (Supplementary Fig. S6e, f). The results demonstrated that folic acid promoted the degradation of RPS15. Furthermore, we performed the co-IP assay to assess the effect of folic acid on the interaction between RPS15 and IGF2BP1 in vitro, which showed that folic acid could inhibit the binding between RPS15 and IGF2BP1 (Fig. 7k, l). These results demonstrate that folic acid had a therapeutic effect on ESCC by directly targeting RPS15/IGF2BP1 interaction, and the effect was augmented by the combination treatment of folic acid with DDP.

## Discussion

Recent studies have refined the comprehension of biological aspects of RPs and reported that some RPLs and RPSs use specific translation pathways to promote tumor progression and drug resistance.^7,33^ In the current study, we screened all RPs using a CRISPR SAM system-based model and found that RPS15 played a key role in the development of ESCC. ESCC is one of the most aggressive types of squamous cell carcinoma and patients with ESCC have a poor prognosis and limited therapeutic options.^34^ RPS15 has recently been reported as a new driver gene in aggressive and chemo-refractory cancer cases.^11,35,36^ However, its role in ESCC remains unknown. Unlike previous studies of RPS15, Ljungström and colleagues observed that somatic mutations in *RPS15* were present in almost 20% of patients with chronic lymphocytic leukemia.^10^ Our previous whole genome sequencing analysis of the ESCC patients failed to identify any mutations of concern in *RPS15*.^37^ Whereas, IHC analysis of TMAs in the current study revealed a higher expression of RPS15 in 504 ESCC tumors compared to that in normal tissues. Kaplan–Meier curves demonstrated that high RPS15 expression in patients with ESCC correlated with a malignant phenotype and poor prognosis. Moreover, increased RPS15 expression was significantly correlated with lymph node metastasis. These results indicate that RPS15 expression in ESCC is a potentially remarkable target that alone is sufficient to affect patient survival.

According to a recent study, the deregulation of RP expression and translation promotes cancer progression.^7^ Therefore, we performed gain and loss of RPS15 function experiments to confirm the activity of RPS15 in promoting ESCC proliferation and metastasis in vitro and in vivo. Morphological measurements, including cell-doubling time, total mitosis events, track speed, and displacement, as well as cell proliferation assays and Boyden Chamber migration and invasion results, indicated that RPS15 promoted the proliferation and motility of ESCC cells. Furthermore, we established a subcutaneous transplantation tumor model, RFP-based lung metastatic mouse model, and popliteal lymph node metastasis model in BALB/c-nude mice using stable RPS15-overexpressing or RPS15-knockout ESCC cell lines. Our results indicated that the high expression of RPS15 may be a driving event in the development of ESCC in vitro and in vivo.

RPS15 is an oncogenic gene and inhibition of its activity may provide an opportunity for an anticancer therapeutic approach. To identify a small molecular inhibitor of RPS15, we screened a marketed drug database. Ultimately, we focused on folic acid, which had a significant growth-suppressing effect on RPS15-overexpressing cells. Folic acid is a small molecule (also known as folate or vitamin B_9_) that plays a vital role in DNA synthesis and replication, cell proliferation, and cell survival.^38–40^ Our current results showed that folic acid in combination with DDP was better at inhibiting the growth of tumor cells compared to that of single-agent treatment in RPS15 high-expression cells. Since many cancer cells overexpress folate receptors, folic acid has long been used as a specific targeting agent for chemotherapy drug delivery.^41,42^ Recent studies have shown that folic acid loading cisplatin improved the cisplatin chemotherapy of ovarian cancer.^43^ As in our results of mass spectrometry (MS) analysis in Fig. 5a, b, we found C1QBP, another RPS15 partner, could bind to RPS15 (data not shown). C1QBP is an evolutionarily conserved protein and has been reported to promote homologous recombination by stabilizing MRE11 and controlling the assembly and activation of MRE11/RAD50/NBS1 complex, and the inhibition of C1QBP enhances the tumor regression with chemotherapy.^44^ RPS15 might interact with C1QBP to promote the DNA damage repair ability of ESCC cells and further promotes chemotherapy tolerance. Therefore, RPS15 inhibition combined with DDP may play a synergistic role by inhibiting the chemotherapy tolerance ability of esophageal squamous cell carcinoma. Thus, further work is needed to definitely establish the potential that the folic acid-based hydrogel for cisplatin may have in improving the outcome of ESCC. Overall, our data demonstrated that folic acid had a therapeutic effect on ESCC by targeting RPS15, which was augmented by the combination with DDP.

Despite the evidence of RPS15 being a cancer driver, the molecular mechanisms of RPS15-linked translation in promoting metastasis remain unclear. Therefore, we used stable RPS15-overexpressing cells and performed RNA-seq and ribosome-seq profiling. Alterations caused by RPS15 overexpression were observed in the MAPK pathway. Protein synthesis and translation assays revealed increased translation efficiencies of MKK6 and p38. Since MKK6 activates p38 through phosphorylation, we selected the p38 inhibitor SB203580 to determine its effects on reducing RPS15 expression in order to test the role of p38 MAPKs in the malignant phenotype of ESCC. Functional assays indicated that SB203580 treatment alone inhibited ESCC cell proliferation and metastasis to a moderate degree, whereas by combining SB203580 and DDP, the proliferation of RPS15-overexpressing ESCC cells was inhibited compared with that in the control cells. Furthermore, the inhibition of SB203580 combined with DDP represented an additional reduction compared with that of SB203580 treatment alone. Western blot analysis indicated that SB203580 treatment combined with DDP significantly reduced the expression of the hallmark of p38 MAPK genes MKK6, p38α, and p-p38α in ESCC cells compared with that of single SB203580 treatment. Our results demonstrated that SB203580 exhibited a therapeutic effect on ESCC by partially blocking RPS15 activity through the p38 MAPK pathway, which was especially notable in combination with DDP.

RPS15 is a key component of the decoding site that neighbors the A-site mRNA codon in the human 40S ribosomal subunit. The oligo-peptide of the C-terminal fragment at positions 120–145 of RPS15 plays an important role in the translation process in eukaryotes.^45^ To investigate the underlying mechanism and the cofactors of RPS15 that activated the p38 MAPK pathway, MS analysis was therefore used to identify the potential interacting proteins of RPS15. Among the proteins identified by MS, we focused on IGF2BP1, IGF2BP2, and IGF2BP3, members of the IGF2BP family. The present study determined the IGF2BP proteins were m^6^A readers that enhanced mRNA stability and translation through their KH domains. Our co-IP assays showed that RPS15 is selectively bound to the KH3-4 domain of IGF2BP1. Furthermore, a molecular docking model and co-IP assays showed that the D82 to A82 mutation destroyed the binding of RPS15 to IGF2BP1. It has been reported that IGF2BP proteins shuttle between ribosomes and non-ribosomes during heat shock and recovery, enhance mRNA storage under stress, and facilitate mRNA translation.^19^ According to IGF2BP1 RIP-seq data, RIP and gene-specific m^6^A assays, IGF2BP1 bound to the 3′-UTR of *MKK6* and *MAPK14* in an m^6^A dependent manner. Our data also demonstrated that RPS15 could enhance the translation of *MAPK14* and *MKK6* mRNA, which are directly bound and recognized by the KH3-4 domain of IGF2BP1.

**Fig. 8:**
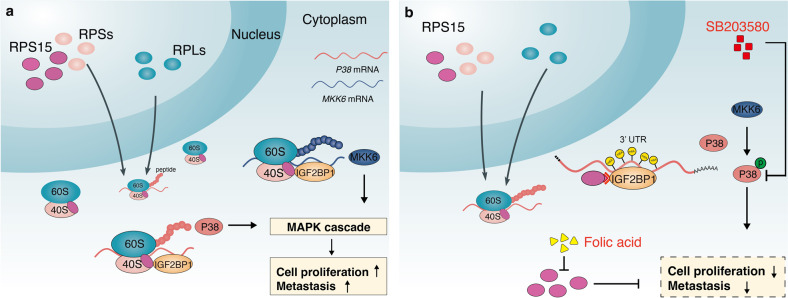
Schematic depicting the contribution of RPS15 and IGF2BP1 to ESCC progression via the p38 MAPK pathway. **a** RPS15 overexpression promotes the proliferation and motility of ESCC cells. Mechanistic investigation revealed that RPS15 interacts with the KH domain of IGF2BP1, which directly binds and recognizes the *MAPK14* and *MKK6* mRNA 3′-UTR, and promotes translation of core p38 MAPK pathway proteins. **b**, RPS15 inhibition with folic acid treatment, IGF2BP1 ablation, or treatment with SB203580 suppresses ESCC proliferation and metastasis via the p38 MAPK signaling pathway

In conclusion, we performed a wide functional analysis of RPS15 to determine the role of the translational machinery in ESCC progression. High expression of RPS15 correlated with the metastasis phenotype and poor prognosis, and RPS15 overexpression promoted the motility and proliferation of ESCC cells. Combining functional experiments in vitro and in vivo and targeted drug virtual screening, folic acid was found to target RPS15 degradation and have a therapeutic effect on ESCC, and the therapeutic effect was augmented by the combination with DDP. Mechanistically, RPS15 interacts with the KH domain of IGF2BP1, which directly binds and recognizes *MAPK14* and *MKK6* mRNA 3′-UTR and promotes the translation of core p38 MAPK pathway proteins. Meanwhile, RPS15 inhibition with folic acid, IGF2BP1 knockout, or treatment with SB203580 will suppress ESCC metastasis and proliferation *via* the p38 MAPK signaling pathway (Fig. 8). Although more evidence regarding the therapeutic effect of RPs should be accumulated, our results suggest that RPS15 has potential as a novel therapeutic target in treating patients with ESCC.

## Materials and methods

### Human ESCC tissue samples

A total of 504 ESCC patients’ tissue samples used in immunohistochemistry were provided by Shanxi Medical University. Informed consent was obtained from all subjects, and this study was approved by the ethical committees of Shanxi Medical University. All the patients had not received prior treatment for their disease. The paired adjacent normal tissue specimen was also obtained for analysis in conjunction with each tumor specimen. Clinical staging of cancer was determined based on the International Union Against Cancer (UICC)/American Joint Commission for Cancer (AJCC) TNM staging system (the eighth edition). Follow-up for all patients was for 2 years. Disease-free survival (DFS) was defined as the time interval after primary surgery during which no sign of cancer is found. The DFS distributions were described by the Kaplan−Meier curves, and statistical significance was calculated using the Log Rank test. Multivariate Cox regression analysis of factors associated with survival of patients with ESCC.

### Immunohistochemistry

Human esophageal cancer tissues were fixed in 4% (v/v) formaldehyde in PBS and then embedded in paraffin and cut into 4 μm sections. Tissue sections were dewaxed in xylene and dehydrated in 95, 85, and 70% alcohol gradient. The slide covered with citrate buffer was heated at 95 °C for 10 min to achieve antigen recovery. Then, 10% goat serum albumin was used to block nonspecific binding, followed by incubation with primary antibodies in a moist chamber at 4 °C overnight. After washing three times with phosphate-buffered saline (PBS), the slides were incubated with a secondary antibody at room temperature for 1 h and washed with PBS. Diaminobenzidine (DAB) was used as a chromogen, and the sections were counterstained with hematoxylin.

### Animal experiments

All animal protocols were approved by the Animal Care and Use Committee of the Chinese Academy of Medical Sciences Cancer Hospital. For subcutaneous xenografting, 1 × 10^6^ KYSE30/KYSE450/KYSE150/KYSE510 cells were subcutaneously implanted into 5-week-old male BALB/c-nude mice (Nanjing Biomedical Research Institute of Nanjing University, Nanjing, China). Mice were fed under normal conditions. Five days later, the mice were randomly assigned to treatment groups and control groups that all received intraperitoneal injections of Folic acid (T0062, TargetMol, 5 mg/kg) and Cisplatin (mab0061, R&D, 5 mg/kg). The tumor lengths and widths were measured using a caliper, and the volume was calculated with the formula 0.5 × length × width^2^. After the tumors had grown for the designated time, all the mice were euthanized, and the tumors were harvested.

For the generation of spontaneous lung metastatic models, 5-week-old BALB/c-nude male mice were injected with 1 × 10^6^ KYSE30 cells stable expressed RPS15 via the tail vein. Three months later, a subset of mice (*n* = 6) was sacrificed, and tumor nodules in the lungs were assessed. For paraffin sections, the lungs were excised and fixed in formalin overnight. Paraffin-embedded lungs were systematically sectioned and stained with hematoxylin and eosin (H&E) staining, and images were taken.

For the metastatic lymph node models, BALB/c-nude mice were injected with 0.1 mL cell suspension containing 1 × 10^6^ RPS15 overexpressed KYSE450 cells into the footpads. Animals were sacrificed at 8 weeks after injection and popliteal lymph nodes were enucleated and paraffin-embedded.

### Cell culture

Human ESCC cell lines were provided by Dr. Yutaka Shimada (Kyoto University, Japan). The HEK293T cell lines were purchased from the American Type Culture Collection (ATCC, VA, USA). ESCC cell lines and HEK293T cells were cultured at 37 °C with 5% CO_2_ in RPMI-1640 medium or Dulbecco’s modified Eagle’s medium (DMEM), respectively, supplemented with 10% FBS. All cells were routinely by short tandem repeat (STR) analysis and regularly tested for mycoplasma contamination.

### Plasmids, lentiviruses, and transduction

The full-length human RPS15 was cloned into the pLenti6-blasticidin and pcDNA3-Flag vector, respectively. The full-length human IGF2BP1 was inserted into the pcDNA3-Myc vector. The mutant plasmids of RPS15 were generated using PCR-based methods with specific primers and subsequently inserted into the pcDNA3-Flag vector. The IGF2BP1 shRNA sequence were cloned into the pSIH-H1 vector.

Lentivirus was produced using HEK293T cells with second-generation packing system pMD2.G (#12 259, Addgene) and psPAX2 (#12 260, Addgene). For lentivirus packaging, 1.5 μg pMD2.G, 4.5 μg psPAX2, together with 6 μg pLenti6/pLenti6-RPS15 or 6 μg pSIH-H1-puro/pSIH-H1-puro-shRNA was co-transfected into HEK293T cells with Hieff Trans Liposomal Transfection Reagent (40802, Yeasen, Shanghai, China). After 48 h, the supernatant was harvested, centrifuged at 1000×*g* for 10 min at 4 °C, and then filtered with a 0.45-μm syringe filter. For viral transductions, 2.5 × 10^5^ cells/well were seeded in six-well culture plates and infected with viruses plus polybrene (8 μg/mL) for 48 h. To obtain stable cell lines, cells were selected for 1-to-2 weeks with 10 μg/mL blasticidin or 1 μg/mL puromycin. Sequences of siRNAs, shRNAs, and sgRNAs are available in Supplementary Table S3.

### Antibodies and reagents

Antibodies against the following proteins were used for Western blot: RPS15 (1:2000, Abcam; ab157193), IGF2BP1 (1:1000, Cell Signaling Technology; 8482), phospho-p38 MAPK (1:1000, Cell Signaling Technology; 9211), p38 MAPK (1:1000, Cell Signaling Technology; 9218), MKK6 (1:1000, Cell Signaling Technology; 8550), β-actin (1:1000, Cell Signaling Technology; 4970), Flag-tag (1:5000, Immunoway; YM3001), Myc-tag (1:5000, Immunoway; YM3203), m^6^A antibody (Thermo; MA5-33030) and rabbit IgG (Millipore; PP64B)-Diquafosol tetrasodium (CAS: 211427-08-6; T7423), Coenzyme A (CAS: 85-61-0; T10857), Folic acid (CAS: 59-30-3; T0062), Epicatechin gallate (CAS: 1257-08-5; T2732), and Methotrexate (CAS: 59-05-2; T1485) were purchased from TargetMol and SB203580 (CAS: 152121-47-6; T1764).

### Immunoprecipitation and Western blot

Total cell lysates were prepared using RIPA lysis buffer with freshly added protease inhibitor cocktail (0 469 315 9001, Roche, Basel, Switzerland) and phosphatase inhibitor cocktail (0 490 684 5001, Roche, Basel, Switzerland) for 20 min on ice. For co-IP assays, IP lysis buffer was used. The protein concentrations were quantified using a BCA assay kit (Thermo Scientific). For immunoprecipitation, equal amounts of protein lysate were incubated overnight at 4 °C with anti-c-Myc magnetic beads (A7470, Sigma) or anti-Flag magnetic beads (M8823, Sigma-Aldrich) for exogenous protein co-IP, and protein A/G magnetic beads (HY-K0202, MCE) or an anti-IGF2BP1 antibody for endogenous protein co-IP. The beads were washed three times with cell lysis buffer and eluted from beads with 2×loading buffer. Protein extracts were resolved on 10% SDS-PAGE, transferred onto PVDF membranes (Merck Millipore, MA, USA), and visualized by chemiluminescence.

### Silver staining and mass spectrometry

Flag-RPS15 was transfected into HEK293T cells, and cell lysates were immunoprecipitated with anti-flag magnetic beads. The beads were washed three times with cell lysis buffer and eluted with 2×loading buffer. The immunoprecipitated protein complexes were resolved on 10% SDS-PAGE, sliver stained with Pierce Silver Stain (24600, Thermo Scientific) and subjected to gel-based LC-MS/MS. MS data were analyzed using MaxQuant software against the UniProtKB/Swiss-Prot human database.

### RNA isolation and quantitative real-time PCR (qRT-PCR)

Total RNA was extracted from cultured cells using TRIzol reagent (Thermo Scientific, MA, USA). RNA was subsequently reverse-transcribed to complementary DNA using a Quantscript RT Kit (KR103, Tiangen, Beijing, China) according to the manufacturer’s instructions. qRT-PCR was carried out using PowerUpTM SYBRTM Green Master Mix (A25742, Applied Biosystems, CA, USA) and the analysis was performed in a StepOnePlus Real-Time PCR system (Applied Biosystems, CA, USA). The relative expression levels of the target genes were standardized as those of the housekeeping gene GAPDH. The qRT-PCR primers are shown in Supplementary Table S2, Supporting Information.

### Gene-specific m^6^A qPCR and RIP-qPCR

Methylated RNA or RNA binding to IGF2BP1 were determined using Magna RIP RNA-Binding Protein Immunoprecipitation Kit (Sigma; 17-700) following the manufacturer’s instructions. One-tenth of fragmented RNA was saved as input control, and further analyzed by qPCR. The related enrichment of m^6^A or IGF2BP1 in each sample was calculated by normalizing to tenfold input. The primers are shown in Supplementary Table S2.

### Transwell assay

The Boyden Chamber (3422/3428, Costar, Corning) precoated with or without Matrigel (354248, Coring, USA) were used to respectively detect the invasion and migration ability of ESCC cells. Cells were seeded into the upper chamber with serum-free medium, whereas a complete medium containing 20% FBS was added to the bottom chamber. After 24 h, the bottom cells were stained with crystal violet. For the inhibitor treatment assays, cells were plated in the upper Chamber in serum-free medium containing p38 MAPK inhibitor SB203580 (T1746, TargetMol, 8 μM), Folic acid (T0062, TargetMol, 10 μM) or DMSO. A complete or conditioned medium containing the corresponding inhibitor was added to the bottom Chamber.

### Live-cell microscopy and quantification

The indicated cell lines were seeded into 96-well plates at a density of 1000–4000 cells per well, depending on the growth rate and design of the experiment. Approximately 24 h later, 10 μM folic acid (T0062, TargetMol), 8 μM p38 MAPK inhibitor SB203580 (T1746, TargetMol), and/or cisplatin were added at the indicated concentrations. Cells were imaged every 3 h using an Incucyte S3 microscope (Essen Bioscience) or Livecyte 419 Phase Focus microscope (Essen Bioscience). Phase-contrast images from the Incucyte S3 microscope were analyzed to evaluate cell proliferation based on cell confluence. Proliferative measurement (cell-doubling time), mitotic measurement (total mitosis events), and kinetic measurements (track speed and displacement) were extracted using Cell Analysis Toolbox (CAT) software available with the Livecyte 419 Phase Focus microscope.

### Virtual screening

The virtual filter available in the Schrodinger software was applied for virtual screening. First, the crystal structure of the RPS15 protein was downloaded from the PDB database (PDB ID 6G4W)^46^ and the P chain was retained. Protein Preparation Wizard was used to optimize the protein structure and the optimized potentials for liquid simulations (OPLS)-2005 force field. Schrodinger sitemap was used to identify three potential small-molecule binding pockets in the RPS15 protein structure. The potential binding pocket with the highest score was defined as the binding pocket based on the Glide score for Receptor Grid Generation (as shown in Fig. 7a).

### RNA-seq and gene expression signature analysis

RNA-seq and gene expression signature analysis were performed as previously described.^47^

### Processing of mRNA-seq and ribo-seq data

Trimmomatic was used to remove adapters and low-quality fragments from the raw reads.^48^ To identify reads originating from rRNAs, Bowtie (version 1.1.3) was used to align the reads to human rRNA sequences downloaded from the National Center for Biotechnology Information Reference Sequence (NCBI RefSeq) database and then excised from the aligned reads.^49^ The remaining reads were then mapped to the human genome (GRCh38) using the STAR (version 2.7.3a) program.^50^ Only uniquely mapped reads with no more than two mismatches were retained.

### Quantification of RPF and mRNA expression

To select high-quality ribosome-protected fragments (RPF), ribo-seq reads with lengths between 26 and 32 nt that mapped to coding sequence regions were used for subsequent quantification analysis. For each gene, mRNA expression and RPF counts were estimated using featureCount (version 1.6.5) software.^51^ Xtail (version 1.1.5) was used to estimate the fold-change in translation efficiency (TE) of each gene between the control cells and RPS15-overexpressing cells.^52^

### Ribosome binding preference

To identify the transcripts preferentially bound by ribosomes in RPS15-overexpressing samples, edgeR Bioconductor package^53^ was used to identify genes with significantly increased RPF counts (*P* < 0.05 and log2 FC >0.45) under conditions of overexpression. ClusterProfiler was then used to perform GO and KEGG enrichment analyses of these genes.^54^

### Statistical analysis

Statistical analyses and graphs were generated using GraphPad Prism version 6.01 software (San Diego, CA, USA). Statistical significance was calculated using an unpaired Student’s *t*-test. Correlation analysis was performed using Pearson’s correlation coefficients. Each experiment was performed at least three times and quantitative data are shown as the mean ± S.D.

## Supplementary information

Supplementary Data

## Data Availability

All data needed to evaluate the conclusions in the paper are present in the paper and/or the Supplementary Materials.
