# Supplementary material for: RPS15 interacted with IGF2BP1 to promote esophageal squamous cell carcinoma development via recognizing m6A modification
Source: Signal Transduct Target Ther. 2023 Jun 2;8:224. doi: 10.1038/s41392-023-01428-1 (PMC10235050; doi:10.1038/s41392-023-01428-1)
Supplement: Supplementary file 1 — Supplementary Data [file 41392_2023_1428_MOESM1_ESM.docx]

Supplementary Materials for

**RPS15 interacted with IGF2BP1 to promote esophageal squamous cell carcinoma development via recognizing m^6^A modification**

Yahui Zhao^1, 7^, Yang Li^1, 7^, Rui Zhu^1^, Riyue Feng^1^, Heyang Cui^2^, Xiao Yu^1^, Furong Huang^1^, Ruixiang Zhang^3^, Xiankai Chen^3^, Lei Li^1,4^, Yinghui Chen^5^, Yuhao Liu^1,4^, Jinhua Wang^6^, Guanhua Du^6^, Zhihua Liu^1*^

Correspondence to: [liuzh@cicams.ac.cn](mailto:liuzh@cicams.ac.cn)

**This PDF file includes:**

Figures. S1 to S6

Tables S1 to S3


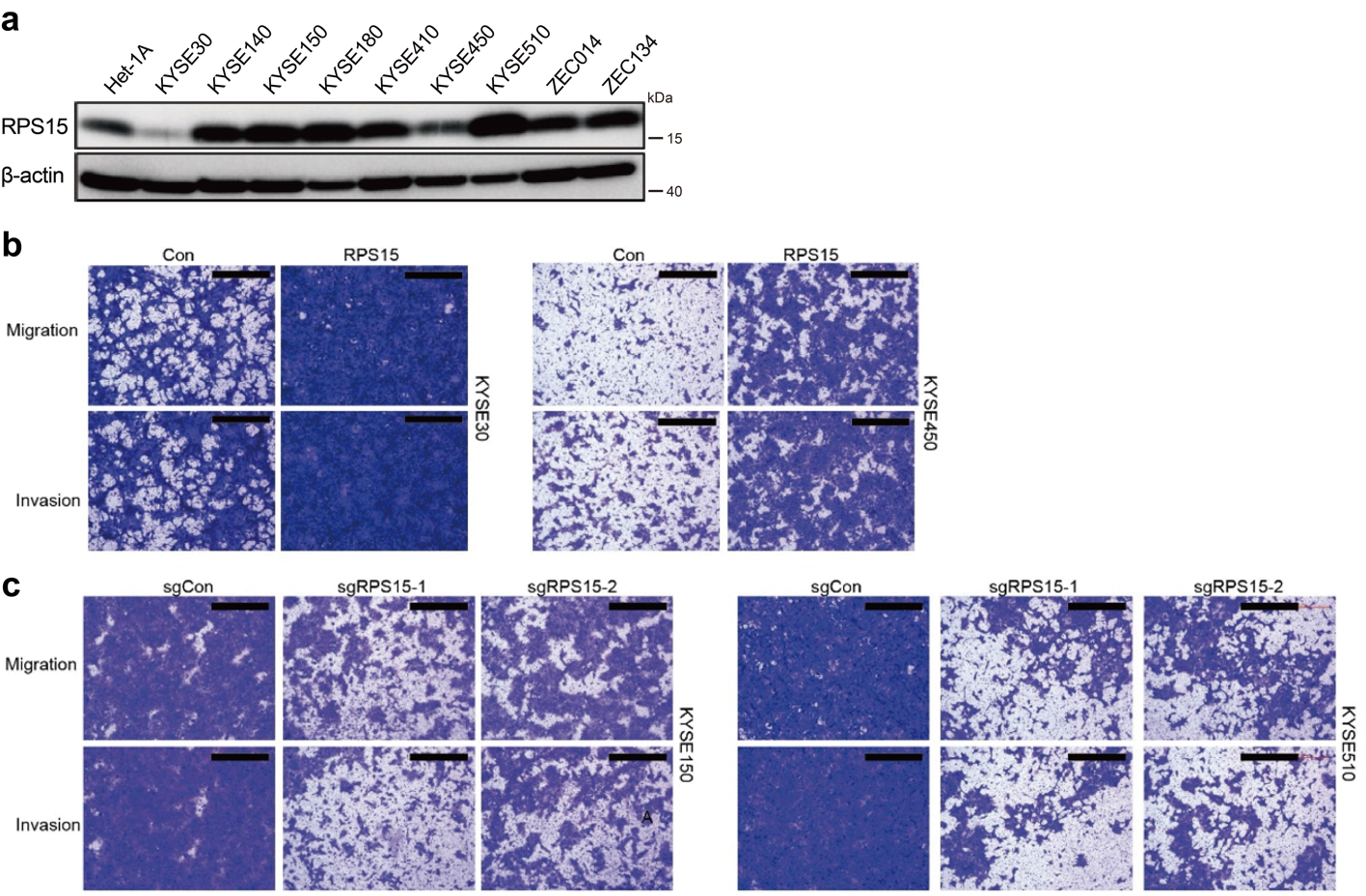


Figure. S1. RPS15 promotes metastasis of ESCC cells *in vitro*.

(a) Expression of RPS15 in nine ESCC cell lines and Het1A cells determined by Western blot.

(b) Representative image of Boyden Chamber migration (upper) and invasion (lower) assays of KYSE30 cells (left) and KYSE450 cells (right) stably transfected with control vector or RPS15-overexpression vector.

(c) Representative image of Boyden Chamber migration (upper) and invasion (lower) assays of KYSE150 cells (left) and KYSE510 cells (right) with or without RPS15 knockout. Scale bar: 500 μm.


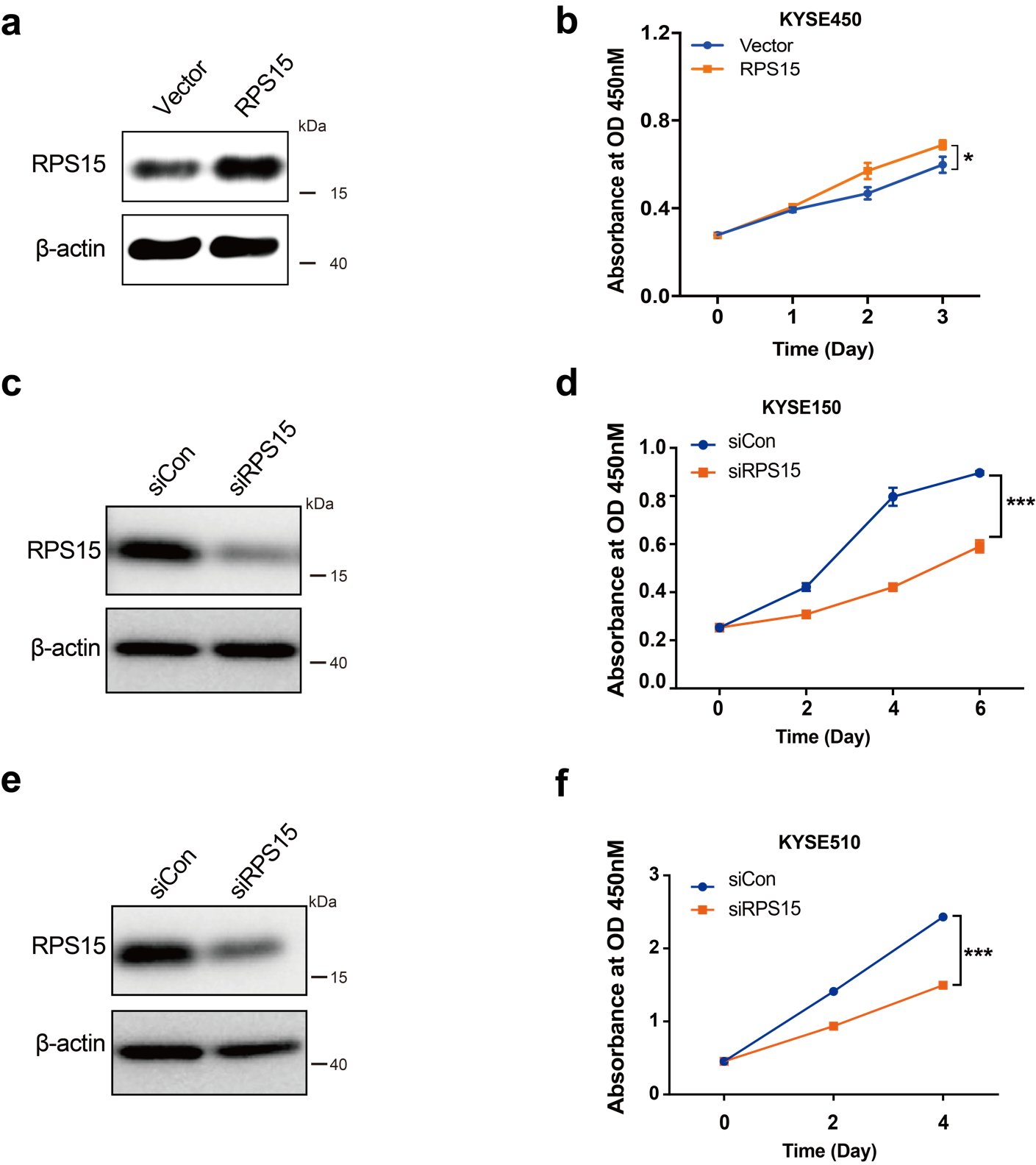


Figure. S2. RPS15 promotes proliferation of ESCC cells in vitro.

(a) Western blot analysis results of RPS15 expression in KYSE450 cells with stably transfected control vector or RPS15-overexpression vector.

(b) Growth curves (measured using CCK8 analyses) of KYSE450 cells stably transfected with control vector or RPS15-overexpression vector for 2 days.

(c and e) Western blot analysis results of RPS15 expression in KYSE150 cells (c) and KYSE510 cells (e) expressing either short interfering RNAs (siRNAs) targeting RPS15 or nontargeting siRNA.

(d and f) Growth curves (measured by CCK8) of KYSE150 cells (d) and KYSE510 cells (f) expressing either siRNAs targeting RPS15 or nontargeting siRNA. The statistical analyses results are shown. Data were analyzed using unpaired t-tests and presented as mean ± S.D.; n≥3. **P*<0.05, ***P*<0.01, ****P*<0.001.


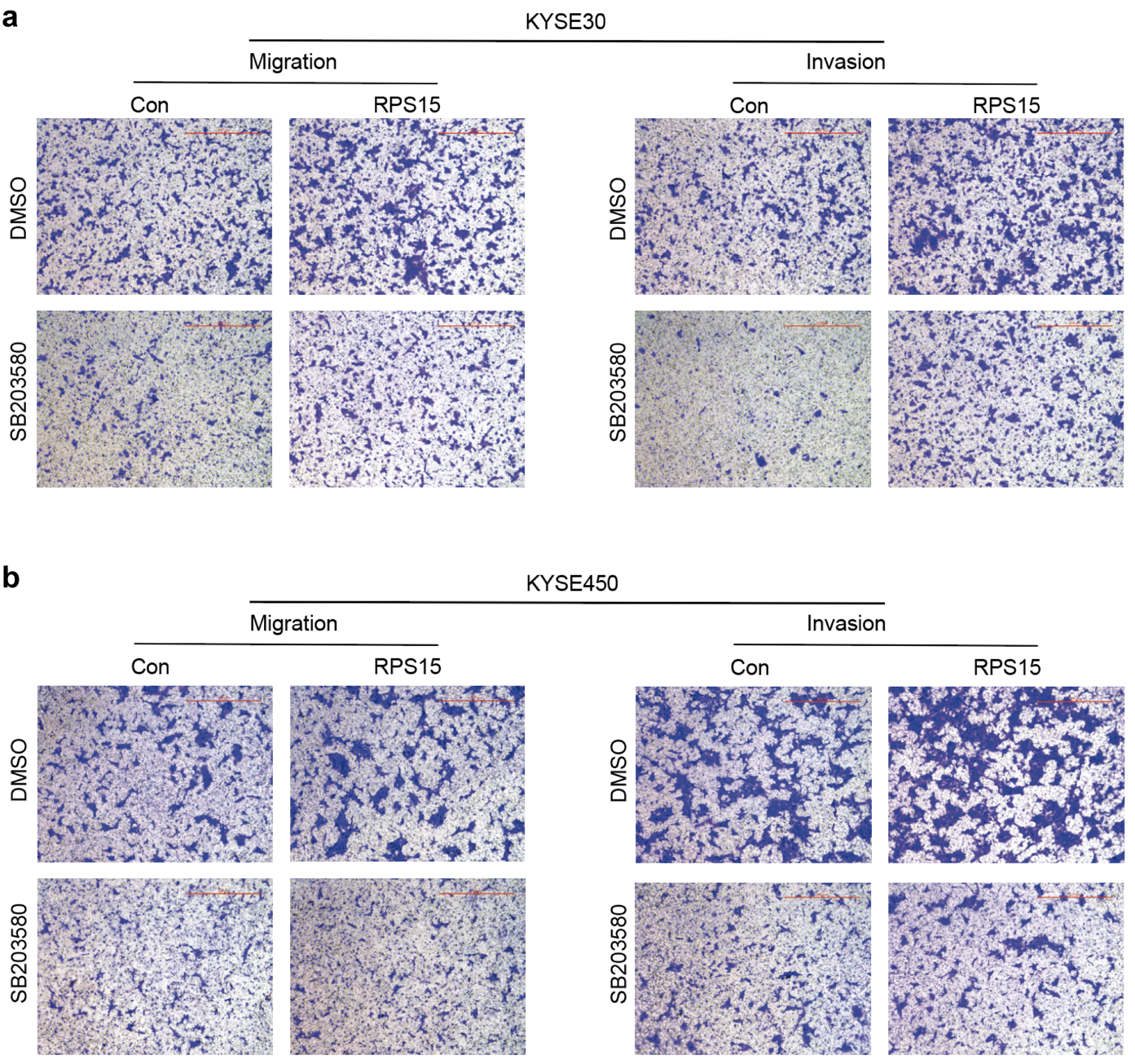


Figure. S3. Inhibition effect of SB203580 on the metastasis ability of RPS15-overexpressed cells.

(a-b) Representative image of Boyden Chamber migration (left) and invasion (right) assays of KYSE30 cells (a) and KYSE450 cells (b) stably transfected with control vector or RPS15-overexpression vector treated with or without SB203580 for 24 hours. Scale bar: 500 μm.

**
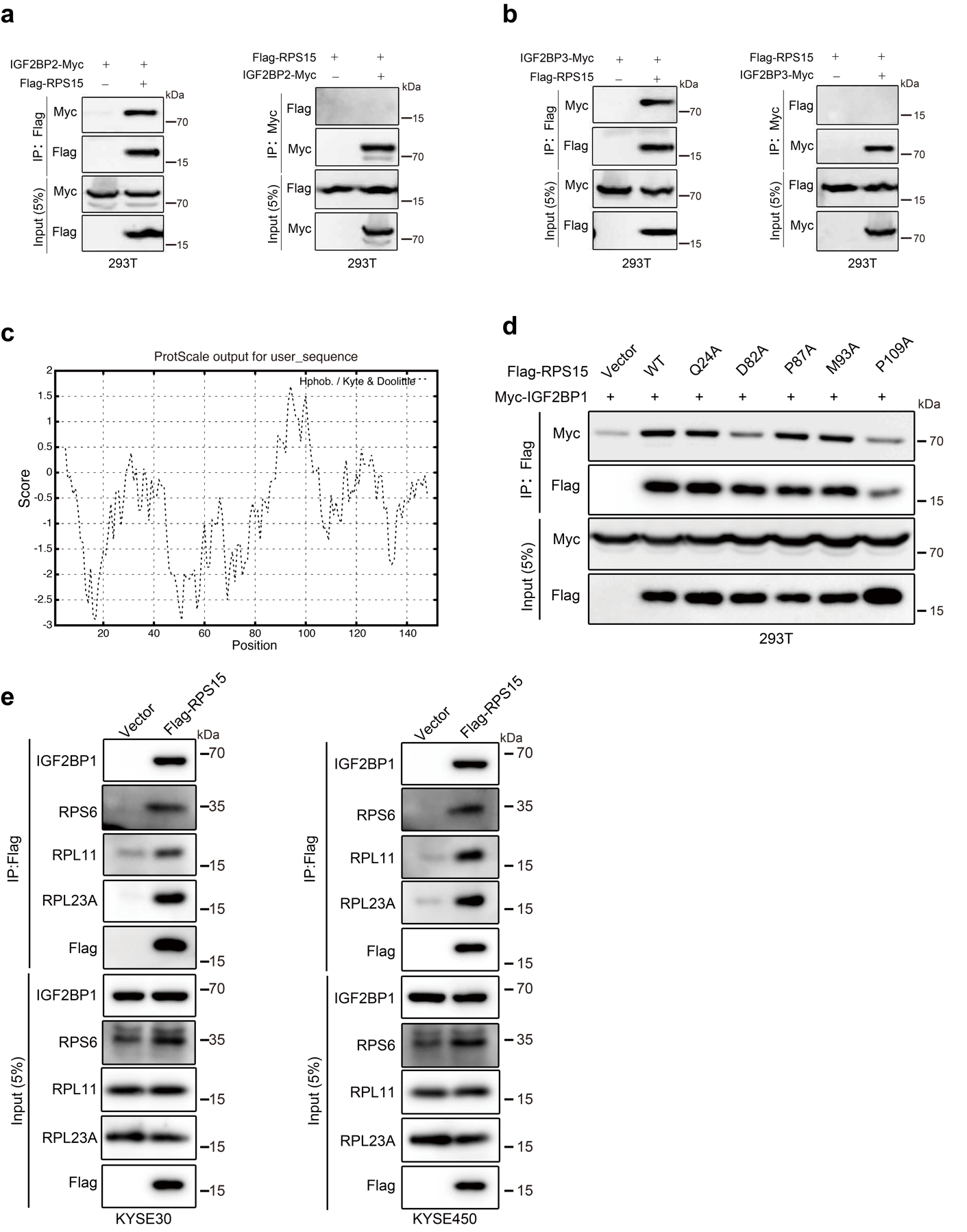
**

**Figure. S4. RS15 specifically binds to IGF2BP1.**

(a) Co-IP assay of RPS15 and IGF2BP2 *in vitro*. Flag-RPS15 and Myc-IGF2BP2 plasmids were transfected into HEK 293T cells. The immunoprecipitates were analyzed by Western blot using anti-FLAG and anti-Myc antibodies.

(b) Co-IP assay of RPS15 and IGF2BP3 *in vitro*. Flag-RPS15 and Myc-IGF2BP2 plasmids were transfected into HEK 293T cells. The immunoprecipitates were analyzed by Western blot using anti-FLAG and anti-Myc antibodies.

(c) Hydrophobicity of RPS15 predicted in ProScale (<https://web.expasy.org/protscale/>).

(d) Co-IP assay to identify the key amino acid sites of RPS15 binding to IGF2BP1. The immunoprecipitates were analyzed by Western blot using an anti-Flag antibody.

(e) Co-IP assay to assess the interaction between RPS15 and main ribosome proteins in Flag-RPS15-overexpressing KYSE30 (left) and KYSE450 (right) cells. The immunoprecipitates were analyzed by Western blot using anti-Flag antibodies.

**
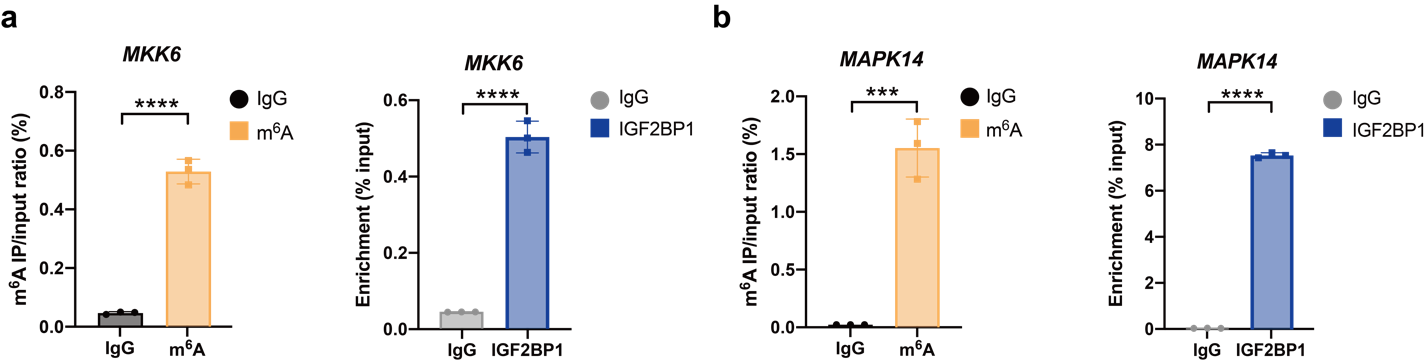
**

**Figure. S5. IGF2BP1 binds the 3’-UTR of *MKK6* and *MAPK14* in an m^6^A dependent manner.**

(a) Enrichment of m6A modification in 3’-UTR region of *MKK6* with Flag-tagged IGF2BP1 in KYSE450 cells (left); RIP-qPCR showing the binding of IGF2BP1 to the 3’-UTR region of *MKK6* (right).

(b) Enrichment of m6A modification in 3’-UTR region of *MAPK14* with Flag-tagged IGF2BP1 in KYSE450 cells (left); RIP-qPCR showing the binding of IGF2BP1 to the 3’-UTR region of *MAPK14* (right). Data were analyzed using unpaired t-tests and presented as mean ± S.D.; n≥3. ****P*<0.001, *****P*<0.0001.

**
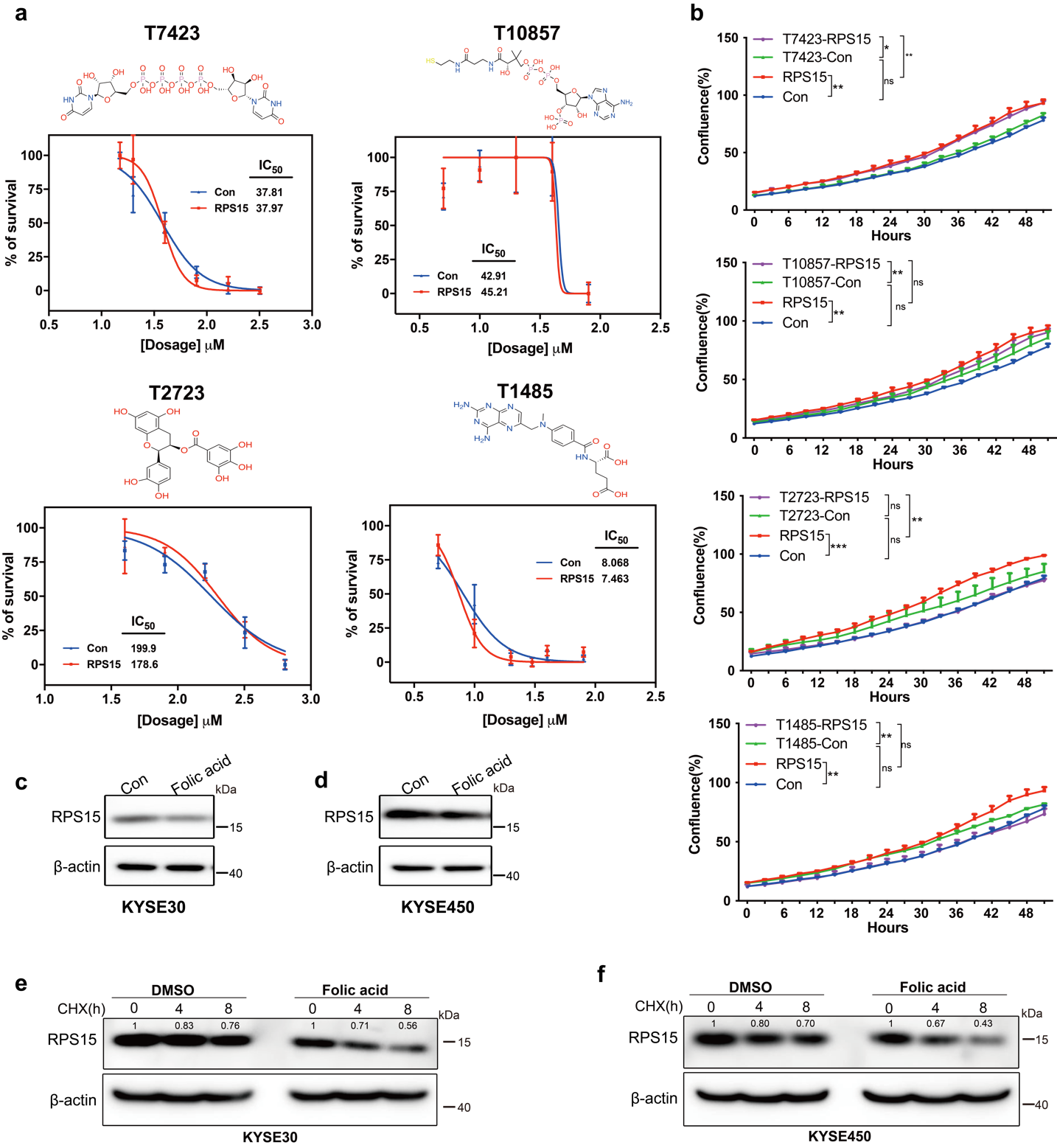
**

**Figure. S6. Inhibition effect of top 5 drugs from docking-based virtual screening.**

(a) Inhibition curves (measured by CCK8 assays) of KYSE30 cells stably transfected with control vector (blue) or RPS15-overexpression vector (red) treated with diquafosol tetrasodium (T7423), coenzyme A (T10857), ketoisophorone (T2723) and methotrexate (T1485) respectively for 24 hours.

(b) Growth curves measured by Incucyte live-cell analyses of KYSE30 cells stably transfected with control vector (blue) or RPS15-overexpression vector (red) treated with (pink) or without (green) four drugs for approximately 2 days. Data were analyzed using unpaired t-tests and presented as mean ± S.D.; n≥3. ***P*<0.01.

(c-d) Western blot detected protein expression of RPS15 in KYSE30 (c) and KYSE450 cells (d), with or without folic acid (T0062, 10 μM) treatment for 24 hours.

(e-f) A cycloheximide (CHX)-chase experiment was performed to compare the rate of RPS15 degradation in KYSE30 (e) and KYSE450 (f) cells with or without folic acid (10 μM) treatment.

**Table S1. List of RPs sg sequences**

| **Name** | **Forward Primer Sequence (5'-3')** | **Reverse Primer Sequence (5'-3')** |
| --- | --- | --- |
| RPLP0—1 | TCCCGGTTCCCTGTGCCACC | GGTGGCACAGGGAACCGGGA |
| RPLP0—2 | TGGAGCTGGAGCTGCGAATT | AATTCGCAGCTCCAGCTCCA |
| RPLP0—3 | AAGGCGCTGCCTCGCGTTGG | CCAACGCGAGGCAGCGCCTT |
| RPLP1—1 | TTTCTCAGCGCCTGCAAATA | TATTTGCAGGCGCTGAGAAA |
| RPLP1—2 | CAGGCGCCTTCAGTGGGTCC | GGACCCACTGAAGGCGCCTG |
| RPLP1—3 | CTCAACAGTAAGAACCAATC | GATTGGTTCTTACTGTTGAG |
| RPLP2—1 | CCGCGAAAAGTGCCTGACGG | CCGTCAGGCACTTTTCGCGG |
| RPLP2—2 | AAGGCGAAGGGCAGCAGTTC | GAACTGCTGCCCTTCGCCTT |
| RPLP2—3 | GCTATAAGAGCAGTCACTTC | GAAGTGACTGCTCTTATAGC |
| RPL3—1 | GACAGAAATTACACACTCTA | TAGAGTGTGTAATTTCTGTC |
| RPL3—2 | TCAAACTTAGTGAAAAGCCG | CGGCTTTTCACTAAGTTTGA |
| RPL3—3 | TTCTGAACGAAAGCTAGGAG | CTCCTAGCTTTCGTTCAGAA |
| RPL3L—1 | CATCAGATAGACAGCCTGAG | CTCAGGCTGTCTATCTGATG |
| RPL3L—2 | GAGGGTCATGACCCAGCCCC | GGGGCTGGGTCATGACCCTC |
| RPL3L—3 | ACTCCTTTCCTCGATGGCCC | GGGCCATCGAGGAAAGGAGT |
| RPL4—1 | TCCGTGTTGGTTCTTAGGCG | CGCCTAAGAACCAACACGGA |
| RPL4—2 | TCGTTTTTACTTTTGTAGTG | CACTACAAAAGTAAAAACGA |
| RPL4—3 | CTAAGAATTATTATTACGTT | AACGTAATAATAATTCTTAG |
| RPL5—1 | CTGTGAGATACCCCGAGCAT | ATGCTCGGGGTATCTCACAG |
| RPL5—2 | GGAACCTGGAGAGCTTGGGC | GCCCAAGCTCTCCAGGTTCC |
| RPL5—3 | TCCGTGCGGTGAGGAGGAGC | GCTCCTCCTCACCGCACGGA |
| RPL6—1 | GCAGGAGGATCCTCTGAGGT | ACCTCAGAGGATCCTCCTGC |
| RPL6—2 | CCCAGCCCTTTGGGAGGCCG | CGGCCTCCCAAAGGGCTGGG |
| RPL6—3 | AAAAGTAGTAAATCCTAGGC | GCCTAGGATTTACTACTTTT |
| RPL7—1 | TTTAGTTCTGGATTCTTTTC | GAAAAGAATCCAGAACTAAA |
| RPL7—2 | AGTTCTTTGCGTCTGCAAGG | CCTTGCAGACGCAAAGAACT |
| RPL7—3 | TGTTGAAAGAGGCTAAATCA | TGATTTAGCCTCTTTCAACA |
| RPL7A—1 | GCGGCGGGGAAGAGAGACTG | CAGTCTCTCTTCCCCGCCGC |
| RPL7A—2 | CTGACTAGGTTTTCGGACCG | CGGTCCGAAAACCTAGTCAG |
| RPL7A—3 | TCCTATAAAGCTAAAAATCG | CGATTTTTAGCTTTATAGGA |
| RPL7L1—1 | TGGCAGGAATCGGGGTTAGC | GCTAACCCCGATTCCTGCCA |
| RPL7L1—2 | GGGACCTCAAGGGCTCACTG | CAGTGAGCCCTTGAGGTCCC |
| RPL7L1—3 | AAAAGTTGCACCGCTTCTTA | TAAGAAGCGGTGCAACTTTT |
| RPL8—1 | CTCCTCCAGGGCCCCCGCGC | GCGCGGGGGCCCTGGAGGAG |
| RPL8—2 | TGTCCTGGTGTGCGTGGGCT | AGCCCACGCACACCAGGACA |
| RPL8—3 | GCACCAGGAGACGCGGCTGA | TCAGCCGCGTCTCCTGGTGC |
| RPL9—1 | GAGGAAATGTCTCTACGCTG | CAGCGTAGAGACATTTCCTC |
| RPL9—2 | TTAGGACTAGCGCAGTGCAG | CTGCACTGCGCTAGTCCTAA |
| RPL9—3 | TTGAGGGATCGCTAACTCCC | GGGAGTTAGCGATCCCTCAA |
| RPL10—1 | ACAGTTAACGGCAAAGCCTA | TAGGCTTTGCCGTTAACTGT |
| RPL10—2 | GCGGGCTTCTCGCGACCATG | CATGGTCGCGAGAAGCCCGC |
| RPL10—3 | CAGACGTATATTTCTGAGAG | CTCTCAGAAATATACGTCTG |
| RPL10A—1 | TGGGCCAACCCCGAGAAGCG | CGCTTCTCGGGGTTGGCCCA |
| RPL10A—2 | GCGTCGCTGGGCCGCTAGGC | GCCTAGCGGCCCAGCGACGC |
| RPL10A—3 | AACTCTGTGGGTTACCGCGC | GCGCGGTAACCCACAGAGTT |
| RPL10L—1 | AAGGTGAGCAGAGTCAGCTG | CAGCTGACTCTGCTCACCTT |
| RPL10L—2 | GACGTCCCAACCAGAGACGC | GCGTCTCTGGTTGGGACGTC |
| RPL10L—3 | GTAGACTCCCCCTGGCGGCC | GGCCGCCAGGGGGAGTCTAC |
| RPL11—1 | GTGGCTAGCACTACCCACAA | TTGTGGGTAGTGCTAGCCAC |
| RPL11—2 | AGGCGACGCTGGCTGAAGAC | GTCTTCAGCCAGCGTCGCCT |
| RPL11—3 | ACTCAAGGACGTGCTGATTC | GAATCAGCACGTCCTTGAGT |
| RPL12—1 | TCTCTAGCTTCAGCGCACCG | CGGTGCGCTGAAGCTAGAGA |
| RPL12—2 | CCGGAGAAGTCTGAGAAGGG | CCCTTCTCAGACTTCTCCGG |
| RPL12—3 | GAACTACCCGAACCCCCTTT | AAAGGGGGTTCGGGTAGTTC |
| RPL13—1 | CCGTCGGTTACTCAGGGCCC | GGGCCCTGAGTAACCGACGG |
| RPL13—2 | CGGCGGACGTCAAGCAAGTC | GACTTGCTTGACGTCCGCCG |
| RPL13—3 | CTCTGATTGGTCAGAGCGCC | GGCGCTCTGACCAATCAGAG |
| RPL13A—1 | GCTGACCGCGGAGGTCCAAC | GTTGGACCTCCGCGGTCAGC |
| RPL13A—2 | GTGCCCGGCGAGAGATCACG | CGTGATCTCTCGCCGGGCAC |
| RPL13A—3 | GAATGTCCGGATTGGACATT | AATGTCCAATCCGGACATTC |
| RPL14—1 | AAGTTGCTTTTTGGCAAAGT | ACTTTGCCAAAAAGCAACTT |
| RPL14—2 | GAAAACATGGCGGAGTCGGC | GCCGACTCCGCCATGTTTTC |
| RPL14—3 | GCACGCGCTCGCAAGCTCAC | GTGAGCTTGCGAGCGCGTGC |
| RPL15—1 | GTCCCCAAGGAGGACCTGAA | TTCAGGTCCTCCTTGGGGAC |
| RPL15—2 | GGAGATGCCGCTGACATCAC | GTGATGTCAGCGGCATCTCC |
| RPL15—3 | AGAACCAGAACTGAGCACCA | TGGTGCTCAGTTCTGGTTCT |
| RPL17—1 | GCCATTTACAAACCACTTTC | GAAAGTGGTTTGTAAATGGC |
| RPL17—2 | TGGCCCTGTACTCCTGAAAG | CTTTCAGGAGTACAGGGCCA |
| RPL17—3 | CAAACCACTTTCAGGAGTAC | GTACTCCTGAAAGTGGTTTG |
| RPL18—1 | AAATTTCCGACCCCACGCTT | AAGCGTGGGGTCGGAAATTT |
| RPL18—2 | GGACCCGGCGGGCCCAGTGT | ACACTGGGCCCGCCGGGTCC |
| RPL18—3 | GGTGAGGAGGCGGGGCTGGC | GCCAGCCCCGCCTCCTCACC |
| RPL18A—1 | TGCGGTCGTGGTGGAACAGG | CCTGTTCCACCACGACCGCA |
| RPL18A—2 | GCGGAACTTTGAGTAGCAGG | CCTGCTACTCAAAGTTCCGC |
| RPL18A—3 | AACGAAGTCTCGCTGAAGAG | CTCTTCAGCGAGACTTCGTT |
| RPL19—1 | TAAAAAAGTACCCCTTCCGT | ACGGAAGGGGTACTTTTTTA |
| RPL19—2 | GGACTATACCACTTTCCCTA | TAGGGAAAGTGGTATAGTCC |
| RPL19—3 | ATCATTTCAGGCCTATATGG | CCATATAGGCCTGAAATGAT |
| RPL21—1 | AAGTGTCAGCTTGTGTTGGC | GCCAACACAAGCTGACACTT |
| RPL21—2 | TTTCCCCTCTTGCGAAAGAA | TTCTTTCGCAAGAGGGGAAA |
| RPL21—3 | GAGAACACAGAAAGTCTGTG | CACAGACTTTCTGTGTTCTC |
| RPL22—1 | TCTCTTGGTTATGTACGATA | TATCGTACATAACCAAGAGA |
| RPL22—2 | AGAGGACGCGTTTTGCATTC | GAATGCAAAACGCGTCCTCT |
| RPL22—3 | TCGCAGTGGGACAGCCCGCC | GGCGGGCTGTCCCACTGCGA |
| RPL22L1—1 | GCTGTGGTTGAACTGACTCT | AGAGTCAGTTCAACCACAGC |
| RPL22L1—2 | TCGGCTTGAACAGCCAGGAG | CTCCTGGCTGTTCAAGCCGA |
| RPL22L1—3 | AGTAGGCCATCGGCTTTCCG | CGGAAAGCCGATGGCCTACT |
| RPL23—1 | GAGGTAGAAGCGCAAGAGAT | ATCTCTTGCGCTTCTACCTC |
| RPL23—2 | CTACGAGCTTAAGAGTAGTT | AACTACTCTTAAGCTCGTAG |
| RPL23—3 | TTTTAGCCCTCTCTGCGAAG | CTTCGCAGAGAGGGCTAAAA |
| RPL23A—1 | AAGGCACTAAAGCTTCATGG | CCATGAAGCTTTAGTGCCTT |
| RPL23A—2 | AACCTAACTACCGTTCCCAG | CTGGGAACGGTAGTTAGGTT |
| RPL23A—3 | TGCGCCACTCCTCCCCTCAA | TTGAGGGGAGGAGTGGCGCA |
| RPL24—1 | CCCTGACAGCTGACTTCCTC | GAGGAAGTCAGCTGTCAGGG |
| RPL24—2 | AGGAGGGCCAGGAGCATCGA | TCGATGCTCCTGGCCCTCCT |
| RPL24—3 | CGCAATTCATCCAGAGGCGC | GCGCCTCTGGATGAATTGCG |
| RPL26—1 | GGCTAGGAGGGGAACTGCGA | TCGCAGTTCCCCTCCTAGCC |
| RPL26—2 | GGAAGGGAGACACTAGGACC | GGTCCTAGTGTCTCCCTTCC |
| RPL26—3 | TTGCAAAACTCCCCTCCTCC | GGAGGAGGGGAGTTTTGCAA |
| RPL26L1—1 | GGATCTCAGTGCCTCATTCC | GGAATGAGGCACTGAGATCC |
| RPL26L1—2 | AAGCAGGCCCTTGTACTCAC | GTGAGTACAAGGGCCTGCTT |
| RPL26L1—3 | GGCCTGCGGTAGAGGCTTAC | GTAAGCCTCTACCGCAGGCC |
| RPL27—1 | TCAGCACCACACAATCAGCG | CGCTGATTGTGTGGTGCTGA |
| RPL27—2 | GCGGGCGGAAGAAGCTCATG | CATGAGCTTCTTCCGCCCGC |
| RPL27—3 | CCAGCAGTAGACAAGAGCAA | TTGCTCTTGTCTACTGCTGG |
| RPL27A—1 | GAGGATTAAAGAGGGCCTCG | CGAGGCCCTCTTTAATCCTC |
| RPL27A—2 | TGCCCACCTGGTGTCTCGCT | AGCGAGACACCAGGTGGGCA |
| RPL27A—3 | TCGGGAGCCGCCCGAGCTTC | GAAGCTCGGGCGGCTCCCGA |
| RPL28—1 | CCGAAAATTCGCTCAACGCT | AGCGTTGAGCGAATTTTCGG |
| RPL28—2 | ATTCGGAACTCTTCGGTTAG | CTAACCGAAGAGTTCCGAAT |
| RPL28—3 | CGGTTCCCGGAATACGCTCT | AGAGCGTATTCCGGGAACCG |
| RPL29—1 | GAAAAAGAAAGGAGTCCTTG | CAAGGACTCCTTTCTTTTTC |
| RPL29—2 | CGTAGCACCGCCTCCACGTC | GACGTGGAGGCGGTGCTACG |
| RPL29—3 | TTGAATAAGACTTAAGAGAC | GTCTCTTAAGTCTTATTCAA |
| RPL30—1 | AAGGTATGAGCAGCCGCGTA | TACGCGGCTGCTCATACCTT |
| RPL30—2 | GATCTGTTGCGTCCAGCTCA | TGAGCTGGACGCAACAGATC |
| RPL30—3 | ACCGCTAGCTAGAATGGCTG | CAGCCATTCTAGCTAGCGGT |
| RPL31—1 | GCGTCCCTTCTCTAAGTCTT | AAGACTTAGAGAAGGGACGC |
| RPL31—2 | GAAGCGGGACCTCAAGCTCG | CGAGCTTGAGGTCCCGCTTC |
| RPL31—3 | GATTCCTTCTGGCTCCGCGA | TCGCGGAGCCAGAAGGAATC |
| RPL32—1 | TTCTACGTAGTCCCAAGGAT | ATCCTTGGGACTACGTAGAA |
| RPL32—2 | CTTTCTGAGCTAATAAGACC | GGTCTTATTAGCTCAGAAAG |
| RPL32—3 | AAATGTATTCTACCTATCCT | AGGATAGGTAGAATACATTT |
| RPL34—1 | CACACTAGCTGTCTGTGTCA | TGACACAGACAGCTAGTGTG |
| RPL34—2 | ACCAACGTGCCGCCCCTCTG | CAGAGGGGCGGCACGTTGGT |
| RPL34—3 | CAGGGAACCACCACGTTGTA | TACAACGTGGTGGTTCCCTG |
| RPL35—1 | AGCACTACAAGGCGGGGCTC | GAGCCCCGCCTTGTAGTGCT |
| RPL35—2 | TATCAGGGGTGCCGTGAATG | CATTCACGGCACCCCTGATA |
| RPL35—3 | ACCACGGCGTTCCTCGGGGG | CCCCCGAGGAACGCCGTGGT |
| RPL35A—1 | TTTCCTAAGTCCTTTCATCT | AGATGAAAGGACTTAGGAAA |
| RPL35A—2 | TGTAAGAGTGCTATTGAATG | CATTCAATAGCACTCTTACA |
| RPL35A—3 | AGGCTGCAAAATGTACTACT | AGTAGTACATTTTGCAGCCT |
| RPL36—1 | AGAACGGCACGCGCTCCCCC | GGGGGAGCGCGTGCCGTTCT |
| RPL36—2 | ACGCGCATGCTCAGGGAGCT | AGCTCCCTGAGCATGCGCGT |
| RPL36—3 | GTCGCGCATGCTCAGCGCAA | TTGCGCTGAGCATGCGCGAC |
| RPL36A—1 | TGGTGGACTCTGCTACGTAG | CTACGTAGCAGAGTCCACCA |
| RPL36A—2 | ACGAAACGTTGAAAGCTGCG | CGCAGCTTTCAACGTTTCGT |
| RPL36A—3 | AGTGTTTTTCCCAGATCCTC | GAGGATCTGGGAAAAACACT |
| RPL36AL—1 | GTTGTCATAACGGTCCCCGC | GCGGGGACCGTTATGACAAC |
| RPL36AL—2 | GGCCGTCTAGGGCCCCTGTA | TACAGGGGCCCTAGACGGCC |
| RPL36AL—3 | AACTGCCCCGCGGCACACCG | CGGTGTGCCGCGGGGCAGTT |
| RPL37—1 | GGGAGTCCCGCCCGCCTTTC | GAAAGGCGGGCGGGACTCCC |
| RPL37—2 | CCATCCGGTCGCCAGAAAAC | GTTTTCTGGCGACCGGATGG |
| RPL37—3 | TCGGGCCTGCTTAGACCCTG | CAGGGTCTAAGCAGGCCCGA |
| RPL37A—1 | GTAAGGCGTCTCTCTCATCC | GGATGAGAGAGACGCCTTAC |
| RPL37A—2 | AACCATAGCAGAAGCATGTC | GACATGCTTCTGCTATGGTT |
| RPL37A—3 | TTTGCGAATCTGGGTAAACC | GGTTTACCCAGATTCGCAAA |
| RPL38—1 | CACTGTTGACTTCGACCACA | TGTGGTCGAAGTCAACAGTG |
| RPL38—2 | AAGAATGGAATGGACCATCC | GGATGGTCCATTCCATTCTT |
| RPL38—3 | GCGGAGGGAGGGCGGCAGCC | GGCTGCCGCCCTCCCTCCGC |
| RPL39—1 | TTCCCTAAGTGCGTTCCCCC | GGGGGAACGCACTTAGGGAA |
| RPL39—2 | GAAGCAAAGCTAACTGCATT | AATGCAGTTAGCTTTGCTTC |
| RPL39—3 | CTAGCACACTGCCGGGCCCC | GGGGCCCGGCAGTGTGCTAG |
| RPL39L—1 | ACACGCAGGAGTCGGACCCA | TGGGTCCGACTCCTGCGTGT |
| RPL39L—2 | AGAGAACTAAGGAAGAATGG | CCATTCTTCCTTAGTTCTCT |
| RPL39L—3 | ATGTGTCACCAAGTTGACTT | AAGTCAACTTGGTGACACAT |
| UBA52—1 | CTCAAGTGACTCGGCGGGCG | CGCCCGCCGAGTCACTTGAG |
| UBA52—2 | CGCCCACCCGCTTCCGGTTG | CAACCGGAAGCGGGTGGGCG |
| UBA52—3 | GCGGACGCAAACACGGGGAG | CTCCCCGTGTTTGCGTCCGC |
| RPL41—1 | GTAGGTTGAGTTAGTTCTTT | AAAGAACTAACTCAACCTAC |
| RPL41—2 | TAACTCGAAAACCAAATACT | AGTATTTGGTTTTCGAGTTA |
| RPL41—3 | TACTAAATCTGGGGGGGAAC | GTTCCCCCCCAGATTTAGTA |
| RPSA—1 | GAACAGCGCACATCTTACTG | CAGTAAGATGTGCGCTGTTC |
| RPSA—2 | TTGAGCTTACAGTGTAGTGA | TCACTACACTGTAAGCTCAA |
| RPSA—3 | CCCATACATGTCGTGTATTA | TAATACACGACATGTATGGG |
| RPS2—1 | GGGCAAGAGCACTCAGAGCC | GGCTCTGAGTGCTCTTGCCC |
| RPS2—2 | CAGGTTGTCTTATAGGAGCT | AGCTCCTATAAGACAACCTG |
| RPS2—3 | TTTCGGGTGACGTAGATTCC | GGAATCTACGTCACCCGAAA |
| RPS3—1 | ACTATGAGAATTGAACTCTA | TAGAGTTCAATTCTCATAGT |
| RPS3—2 | TGCCTACATTCTTCTTTCCC | GGGAAAGAAGAATGTAGGCA |
| RPS3—3 | GGAAAGGGGCTTCATCCAGC | GCTGGATGAAGCCCCTTTCC |
| RPS3A—1 | AGGCTGCGCTGTGTAGACCA | TGGTCTACACAGCGCAGCCT |
| RPS3A—2 | GCGTCGGTGGCCTGACGTGC | GCACGTCAGGCCACCGACGC |
| RPS3A—3 | TGCGACATCGCCATCGTGTG | CACACGATGGCGATGTCGCA |
| RPS4X—1 | GGAGGGAGGAAATCGCCCTT | AAGGGCGATTTCCTCCCTCC |
| RPS4X—2 | CCTTATTCTGTGCTAGGTGC | GCACCTAGCACAGAATAAGG |
| RPS4X—3 | GAGACACCCTCTTTCCGCGG | CCGCGGAAAGAGGGTGTCTC |
| RPS4Y1—1 | CCCCGTCAAGTTGGCTGAGC | GCTCAGCCAACTTGACGGGG |
| RPS4Y1—2 | AGGCACAACAAAAAGTCCTG | CAGGACTTTTTGTTGTGCCT |
| RPS4Y1—3 | AGTTTCTGATACTGATTTAC | GTAAATCAGTATCAGAAACT |
| RPS4Y2—1 | CATAGGTGTCAGTTATGCCA | TGGCATAACTGACACCTATG |
| RPS4Y2—2 | TCCCCTCAGGTCGGCTAAGC | GCTTAGCCGACCTGAGGGGA |
| RPS4Y2—3 | AAGCACAACAAAAAGTCCCG | CGGGACTTTTTGTTGTGCTT |
| RPS5—1 | AGCGGCCCTAGAAGCGTGCC | GGCACGCTTCTAGGGCCGCT |
| RPS5—2 | ATTGGTCTATAGGGACACGA | TCGTGTCCCTATAGACCAAT |
| RPS5—3 | CCCATTCCGTCTAGCCCAAA | TTTGGGCTAGACGGAATGGG |
| RPS6—1 | GCCTGGGAGGTAAAGTCGGT | ACCGACTTTACCTCCCAGGC |
| RPS6—2 | ACTTCCGGGCGAGATTAATC | GATTAATCTCGCCCGGAAGT |
| RPS6—3 | TGGGTCTGAGGTTGTGCCAG | CTGGCACAACCTCAGACCCA |
| RPS7—1 | GGAACCTTGGGAGCCGGTAC | GTACCGGCTCCCAAGGTTCC |
| RPS7—2 | GGTGCCGTTTCCGAGCGTTC | GAACGCTCGGAAACGGCACC |
| RPS7—3 | TGGTAACGCTTTTCCGCCCT | AGGGCGGAAAAGCGTTACCA |
| RPS8—1 | TGCTGACCTCTGCCGACCCC | GGGGTCGGCAGAGGTCAGCA |
| RPS8—2 | GGCGACTCGCCTCCGACCCT | AGGGTCGGAGGCGAGTCGCC |
| RPS8—3 | CGAGCCTAGGAGGGTCTCGC | GCGAGACCCTCCTAGGCTCG |
| RPS9—1 | GTTGGCTGGCTGAGAAGCAG | CTGCTTCTCAGCCAGCCAAC |
| RPS9—2 | CGTGCTAACCTGGGAGGACT | AGTCCTCCCAGGTTAGCACG |
| RPS9—3 | GGTTTTTTCCGGCCAGGGAG | CTCCCTGGCCGGAAAAAACC |
| RPS10—1 | ACGCACTAGGAAACCGCCGC | GCGGCGGTTTCCTAGTGCGT |
| RPS10—2 | CCTCAGGCGGTCAGGCTCCG | CGGAGCCTGACCGCCTGAGG |
| RPS10—3 | GTCAGATCCGCCAGTTCTTC | GAAGAACTGGCGGATCTGAC |
| RPS11—1 | CGGTGTCTCGCGAGAGTCTT | AAGACTCTCGCGAGACACCG |
| RPS11—2 | TAATCGCGCCCGGCGTTGTG | CACAACGCCGGGCGCGATTA |
| RPS11—3 | GGGCACTGTGAAGGACTGAC | GTCAGTCCTTCACAGTGCCC |
| RPS12—1 | GCACTCGCCAAGGACCGGCC | GGCCGGTCCTTGGCGAGTGC |
| RPS12—2 | GCGAGTTTTCTATCTCTGCC | GGCAGAGATAGAAAACTCGC |
| RPS12—3 | TCCGGATTGAGGCGGTTCTG | CAGAACCGCCTCAATCCGGA |
| RPS13—1 | TCGTCAATCCAGCCTCATCC | GGATGAGGCTGGATTGACGA |
| RPS13—2 | ACCAAAGACCGATGCATGCC | GGCATGCATCGGTCTTTGGT |
| RPS13—3 | GCGACGCTGAAATGCATTTC | GAAATGCATTTCAGCGTCGC |
| RPS14—1 | ACTTAAGGGAGAGCGAAAAG | CTTTTCGCTCTCCCTTAAGT |
| RPS14—2 | GGGCTGGTTGTCGCTGCGAG | CTCGCAGCGACAACCAGCCC |
| RPS14—3 | CTCTTTGGGAGGAATAATGC | GCATTATTCCTCCCAAAGAG |
| RPS15—1 | CTGCAGAGCCGCTTCCTCCA | TGGAGGAAGCGGCTCTGCAG |
| RPS15—2 | ATTAGAGCAGCCGCCGCCTG | CAGGCGGCGGCTGCTCTAAT |
| RPS15—3 | GAGGGCAAAACACAGGCTGC | GCAGCCTGTGTTTTGCCCTC |
| RPS15A—1 | GGCGGAGCCTCCTCAGGGGT | ACCCCTGAGGAGGCTCCGCC |
| RPS15A—2 | GAAGGACGAGGGTGAGGGAA | TTCCCTCACCCTCGTCCTTC |
| RPS15A—3 | TCGATGGAATCGACCTCCCC | GGGGAGGTCGATTCCATCGA |
| RPS16—1 | GGGCCTGGGAGTGTCGTGTG | CACACGACACTCCCAGGCCC |
| RPS16—2 | GGCGGGCTCTGTGGGCGGAA | TTCCGCCCACAGAGCCCGCC |
| RPS16—3 | AGGCCCTTAGGCGCTCAGAG | CTCTGAGCGCCTAAGGGCCT |
| RPS17—1 | AAGGGAGGAGCCGTCGGGGG | CCCCCGACGGCTCCTCCCTT |
| RPS17—2 | GCTAGAGCACATCGCACGGG | CCCGTGCGATGTGCTCTAGC |
| RPS17—3 | CTCCCCCATCTGATTTTTAA | TTAAAAATCAGATGGGGGAG |
| RPS18—1 | ATACTCTCATAAATCCAGCC | GGCTGGATTTATGAGAGTAT |
| RPS18—2 | TAAGAGAACAGCGAAGGTTC | GAACCTTCGCTGTTCTCTTA |
| RPS18—3 | TTACAGTTCGTAAGGTTCAT | ATGAACCTTACGAACTGTAA |
| RPS19—1 | ATCTTCCGTCCTTCACCGCC | GGCGGTGAAGGACGGAAGAT |
| RPS19—2 | GGCCGGAAGTGGGAGTAGTA | TACTACTCCCACTTCCGGCC |
| RPS19—3 | GGAACTGTGACAGGGAACCT | AGGTTCCCTGTCACAGTTCC |
| RPS20—1 | GGCCACCTGGTCGAGAGAAC | GTTCTCTCGACCAGGTGGCC |
| RPS20—2 | ACCTGAGAAACTCCTCTGTC | GACAGAGGAGTTTCTCAGGT |
| RPS20—3 | GGCGCCCCCTGTCCGCACGG | CCGTGCGGACAGGGGGCGCC |
| RPS21—1 | TTGAGGGTCGTTGCCGTGTG | CACACGGCAACGACCCTCAA |
| RPS21—2 | GGACCCCAGCCTGCTGGCCC | GGGCCAGCAGGCTGGGGTCC |
| RPS21—3 | GCTTCCGGCAAGGAGGTTCT | AGAACCTCCTTGCCGGAAGC |
| RPS23—1 | CCCCTTTTTCTTTTGAATGC | GCATTCAAAAGAAAAAGGGG |
| RPS23—2 | ATTGGTAGTCCATGTTCCTC | GAGGAACATGGACTACCAAT |
| RPS23—3 | TCAGAGATATCTAATTTGCA | TGCAAATTAGATATCTCTGA |
| RPS24—1 | CTAGTCGGCTCGAGTTGTTC | GAACAACTCGAGCCGACTAG |
| RPS24—2 | GAGTTGTTCTGGTTCTGGAT | ATCCAGAACCAGAACAACTC |
| RPS24—3 | CCGACTAGGTTTACGCAAGC | GCTTGCGTAAACCTAGTCGG |
| RPS25—1 | GTATTTAGGCGGTGCGGCTT | AAGCCGCACCGCCTAAATAC |
| RPS25—2 | AGCGCTCGCCCTCTAGCCTG | CAGGCTAGAGGGCGAGCGCT |
| RPS25—3 | GTCACAACTAGCGCAGACTC | GAGTCTGCGCTAGTTGTGAC |
| RPS26—1 | AATTGTAGTGCGAAGCCAAG | CTTGGCTTCGCACTACAATT |
| RPS26—2 | TTGCCAGACGCTGGACCACA | TGTGGTCCAGCGTCTGGCAA |
| RPS26—3 | CTCGTTATTAGAGGCCAGAG | CTCTGGCCTCTAATAACGAG |
| RPS27—1 | AGTTAAAGACCTTCCGAAAA | TTTTCGGAAGGTCTTTAACT |
| RPS27—2 | ATATGCGTCACCATGTTGAG | CTCAACATGGTGACGCATAT |
| RPS27—3 | GCTCCCTTGTCTCCCCACTG | CAGTGGGGAGACAAGGGAGC |
| RPS27A—1 | TATGTATGGGGTGCTTCTTC | GAAGAAGCACCCCATACATA |
| RPS27A—2 | TGGATCTGAGAGGAAGTTTT | AAAACTTCCTCTCAGATCCA |
| RPS27A—3 | GAATTTCCAAATTTGGGCAT | ATGCCCAAATTTGGAAATTC |
| RPS27L—1 | TGGGGAGCGGCCTCGGGCTT | AAGCCCGAGGCCGCTCCCCA |
| RPS27L—2 | GCCAAGTCCCGTGGGTCTAA | TTAGACCCACGGGACTTGGC |
| RPS27L—3 | CTGGAACGCTCACCATCCCC | GGGGATGGTGAGCGTTCCAG |
| RPS28—1 | CCGCCACCCGTCTCATCCAG | CTGGATGAGACGGGTGGCGG |
| RPS28—2 | CCCAGCGGTGGAGACGTCAC | GTGACGTCTCCACCGCTGGG |
| RPS28—3 | GGCTGCGCCCTTCAGTATCG | CGATACTGAAGGGCGCAGCC |
| RPS29—1 | GTATGGTGACGTCATCAACT | AGTTGATGACGTCACCATAC |
| RPS29—2 | CAGAATAGCACTAGAAGCTG | CAGCTTCTAGTGCTATTCTG |
| RPS29—3 | CGGTATCCGACCGCCGGGCG | CGCCCGGCGGTCGGATACCG |
| AU—1 | GCTGGCGTAGCAGGTAAAGA | TCTTTACCTGCTACGCCAGC |
| AU—2 | CCATGTTCCGGGCTACGCTG | CAGCGTAGCCCGGAACATGG |
| AU—3 | CAGACAGTTGCGCGCACAGA | TCTGTGCGCGCAACTGTCTG |

Table S2. List of qRT-PCR primer sequences

| **Primer name** | **Forward Primer Sequence (5'-3')** | **Reverse Primer Sequence (5'-3')** |
| --- | --- | --- |
| MKK6 | GAAGCATTTGAACAACCTCAGAC | CCTGGCTATTTACTGTGGCTC |
| MAPK14 | TCAGTCCATCATTCATGCGAAA | AACGTCCAACAGACCAATCAC |
| RPLP0 | CAGATTGGCTACCCAACTGTT | GGAAGGTGTAATCCGTCTCCAC |
| RPLP1 | AGCCTCATCTGCAATGTAGGG | TCAGACTCCTCGGATTCTTCTTT |
| RPLP2 | TTGGACAGCGTGGGTATCG | CCAGCAGGTACACTGGCAA |
| RPL3 | CTACCATCACCGCACTGAGAT | GGTCACTTCACCATAGTGGACA |
| RPL3L | CCCCACTACGGGGAAGTGA | GAGGGACTTTCTCAGCGTAATG |
| RPL4 | TGTTTGCACCAACCAAAACCT | GCAGAACAGATGGCGTATCGT |
| RPL5 | GAGGCTTGTCTATCCCTCACA | GTGCTTCCGATGTACTTCTGC |
| RPL6 | GGTCCTCAATCGAGTTCCTCT | CGCTGCTCCGTAATCTCATATT |
| RPL7 | CAAGGCTTCGATTAACATGCTGA | GCCATAACCACGCTTGTAGATT |
| RPL7A | GTGGTGAATCCCCTGTTTGAG | CAAAGCGGGTGAGGTCTCTT |
| RPL7L | TTTGTTGTACGCATCGAAAGGA | GGTTCCACTATACGCAGCATT |
| RPL8 | AAGGGCATCGTCAAGGACATC | CAGCTCCGTCCGCTTCTTAAA |
| RPL9 | GCACAGTTATCGTGAAGGGC | TTACCCCACCATTTGTCAACC |
| RPL10 | GTGGGGCTTCACCAAGTTCAA | CCACGACTGGGGATGTACT |
| RPL10A | CACACAACGAAAACATGGTGG | CAAGCTCATCGTCTGTCATCTT |
| RPL10L | GCCGTACCCAAAATCTCGTTT | CCACCGAGTGGGAACTCAT |
| RPL11 | GGGAACTTCGCATCCGCAA | CGCACCTTTAGACCCTTCTCC |
| RPL12 | AAAGCCCTCAAGGAACCACC | GCATCTGTCGAGCAATGTTGAC |
| RPL13 | TCAAAGCCTTCGCTAGTCTCC | GGCTCTTTTTGCCCGTATGC |
| RPL13A | GCCATCGTGGCTAAACAGGTA | GTTGGTGTTCATCCGCTTGC |
| RPL14 | GACCTTGCACTCAAGTGAGGA | CTTGTCGGACATACTTCTGGTG |
| RPL15 | AAGGGTGCAACTTACGGCAA | CTCCTCTGCAACGGACTGAA |
| RPL17 | GAACACTCGTGAAACTGCTCA | AACGTCGGAATGGTACACACT |
| RPL18 | ATGTGCGGGTTCAGGAGGTA | CTGGTCGAAAGTGAGGATCTTG |
| RPL18A | ATCTTTGCGCCTAATCATGTCG | CCACAGTAGACAATCTCCCCT |
| RPL19 | AAAACAAGCGGATTCTCATGGA | TGCGTGCTTCCTTGGTCTTAG |
| RPL21 | TAAGCACTCTAAGAGCCGAGAT | GCGCTTTAGTTGAACCCAGGTA |
| RPL22 | AAAGTGAACGGAAAAGCTGGG | TCACGGTGATCTTGCTCTTGC |
| RPL22L1 | GCAATTTCTACGGGAGAAGGTT | ACTCGAAGCCAATCACGAAGA |
| RPL23 | ATCAAGGGACGGCTGAACAG | TCGTTGTCGAATGACCACTGC |
| RPL23A | GGGCAAGACTCCTGTTCT | CCTGGGCATTGGTTTCAT |
| RPL24 | CGCCGAGCAGTCAAATTCC | TCGTTGAGCCTTTCTAACTTCAG |
| RPL26 | GACTTCCGACCGAAGCAAGAA | TGCACCCGTTCAATGTAGATAAC |
| RPL26L1 | AAATTGGCAAGGTAGTCCAGG | ACTTGGCTTTGCGTTCAAGAA |
| RPL27 | TGGCTGGAATTGACCGCTAC | CCTTGTGGGCATTAGGTGATTG |
| RPL27A | TGAGGAAGACCCGGAAACTTA | GCCTGGGTGGTATTTGTCGAA |
| RPL28 | GCATCTGCAATGGATGGTCG | GTCCGTTGTAGCGGAAGGAA |
| RPL29 | CAGTCCCGAAAATGGCACAGA | GGCTTTACGAGGGCCTTGATA |
| RPL30 | GCTGGAGTCGATCAACTCTAGG | CCAATTTCGCTTTGCCTTGTC |
| RPL31 | CTCGGGCACTCAAAGAGATTC | CGGATTCGGTATGGCACATTC |
| RPL32 | TTAAGCGTAACTGGCGGAAAC | AAACATTGTGAGCGATCTCGG |
| RPL34 | GTTTGACATACCGACGTAGGC | GCACACATGGAACCACCATAG |
| RPL35 | AGCTCTCTAAGATCCGAGTCG | GAACACGGGCAATGGATTTCC |
| RPL35A | TTGAAGGTGTTTACGCCCGAG | TGCTTCGGAATTTGGCACGA |
| RPL36 | CACCAAGTTCGTGCGGGACA | TCAGAGCGGGCACCAGACAG |
| RPL36A | CTAAAACCCGCCGGACTTTCT | CTTCCTGTCATAACGCCGCTT |
| RPL36AL | AGATTGTGCTAAGGCTGGAA | TGAGGTGGGACTACACTAAA |
| RPL37 | GCAGAAGCGAGATGACGAAG | AGCCCAGTCCCTAAACCTAC |
| RPL37A | ATTGAAATCAGCCAGCACGC | AGGAACCACAGTGCCAGATCC |
| RPL38 | CATGATCTCAGTCGGCAAAT | CAAGCCAATGAAAGAGGTGT |
| RPL39 | CCCTCCTCTTCCTTTCTCCG | ACTGAATCCAGCCAACCAAC |
| RPL39L | ACACGCAGACCTGAGGGAGC | TGGCGAGAAACAGAGTCAACCA |
| UBA52(RPL40) | AAGACAAGGAGGGTATCCCAC | TGTTGTAGTCTGAGAGAGTGCG |
| RPL41 | AGTGGAGGAAGAAGCGAATG | TGTTTATGAGCAAGGTGGGT |
| RPSA | GTGGCACCAATCTTGACTTCC | GCAGGGTTTTCAATGGCAACAA |
| RPS2 | GGCCTCTCTCAAGGATGAGGT | GTCCCCGATAGCAACAAATGC |
| RPS3 | AGAGGAAGTTTGTCGCTGATG | GCACCTCAACTCCAGAGTAGC |
| RPS3A | GGCAAGAACAAGCGCCTTAC | CAGGTGCTTTCACATCATACCAA |
| RPS4X | GGCAAGGTCCGAACTGATATAAC | GGAAATTCTCTCCCGTCTTGTCA |
| RPS4Y1 | TGACAAACTAACGGGTGTATT | TTATCTTGCCAGTCCCTAAA |
| RPS4Y2 | ACAGTGGAAGAGGCAAAGTA | TCCCAGTCCCTAAATCAATC |
| RPS5 | AGGATGACCGAGTGGGAGAC | GGCAGGTACTTGGCATACTTCT |
| RPS6 | AGGGTTATGTGGTCCGAATCA | TTGGTCTGTAACAGGAATGCC |
| RPS7 | CCAAGTCCGGCTAGTACGC | TCGAGTTGGCTTAGGCAGAAT |
| RPS8 | TACAAACCGAACCGTGAATC | TACGAGTACAACACTCTGAGCC |
| RPS9 | TGAAGCTGGATTACATCCTGGG | CTGCTTGCGGACCCTGATATG |
| RPS10 | TCTCCGTGATTACCTTCATCTGC | TCCAGACCTTTAGGCCGAGG |
| RPS11 | TTCAGACTGAGCGTGCCTAC | GCCCTCAATAGCCTCCTTGG |
| RPS12 | TTGTGTGCTTGCATCCAACTG | GGCCTACCCATTCTCCTAGTTTC |
| RPS13 | AAGTACGTTTTGTGACAGGCA | CGGTGAATCCGGCTCTCTATTAG |
| RPS14 | AAAGGCAGACCGAGATGAATCC | TGATGTGTAGGGCGGTGATAC |
| RPS15A | GTGCAACTCAAAGACCTGGAA | TCCCTCCTGTGTGTTTTCGTC |
| RPS16 | AGGAGCGATTTGCTGGTGTAG | GAGATGGACTGACGGATAGCATA |
| RPS17 | AGGAGATCGCCATTATCCCCA | ATGCGTGACATAACCTGCTATC |
| RPS18 | ATCACCATTATGCAGAATCCACG | GACCTGGCTGTATTTTCCATCC |
| RPS19 | GCCTTCCCAGGTCAAACAGT | CTCACAGCCATCCTTTCGTT |
| RPS20 | AACAAGCCGCAACGTAAAATC | ACGATCCCACGTCTTAGAACC |
| RPS21 | GGCGAGTTCGTGGACCTGTA | GGCCATTAAACCTGCCTGTG |
| RPS23 | GTACCCAATGACGGTTGCTTG | CAGCATGACCTTTGCGACC |
| RPS24 | TGCCTAAGACAGAAATTCGGGA | TCTTGCGTTCCTTTCGTTGCT |
| RPS25 | AAGGACGCTGGAAAGTCGG | CTTTGCCTTTGGACCACTTCT |
| RPS26 | CTGAAGCGAGCGTCTTCGAT | TGAATTGCACAACTCACACAGTA |
| RPS27 | ATGGATGTGAAATGCCCAGGAT | GGCTGGCAGAGGACAGTGGA |
| RPS27A | AAAATTAGTCGCCTTCGTCG | ACAGAACCACTGGGTCACAT |
| RPS27L | TGGGCTCCATACAGTTTCTC | GGCACTACATTCCCTTTGAT |
| RPS28 | CACGAGCCGATCCATCATCC | GAACCCGACATCCAAGACCC |
| RPS29 | AGAGCAAGATGGGTCACCAG | AGACACGACAAGAGCGAGAA |
| FAU(RPS30) | GTCGCCAATATGCAGCTCTT | TGCTACTTCCAGGGTAGTCAGG |
| FAU(RPS30) | CCCGGAAGATCAAGTCGTGC | CACCTTAGGAGTCTGACCTCTC |
| MKK6 3’UTR | GGGAGATAAGAGCGAGGTA | GAGGTTCAGATAGGCAGA |
| MKK14 3’UTR | GCGTAGACTTGACAACATC | AGGTGAAGGGTAAGCAGAG |

Table S3. siRNAs, sgRNAs and shRNAs sequences

| **Name** | **Sequence (5'-3')** | **Purpose** |
| --- | --- | --- |
| RPS15-si#1 | CGGAAGTGGTGAAGACGCA | siRNA |
| RPS15-si#2 | CAGAAGAAGAAGCGGACCT | siRNA |
| RPS15-si#3 | AGGTGGAGATCAAGCCCGA | siRNA |
| RPS15-si#4 | CCTACAAGCCCGTAAAGCA | siRNA |
| IGF2BP1-sh1 | ACGCTTAGAGATTGAACATTC | shRNA |
| IGF2BP1-sh2 | GATGGATGCTACGAGTATAAA | shRNA |
| Non-targeting | AGTCTTAATCGCGTATAAGGC | shRNA |
